# Myc stimulates cell cycle progression through the activation of Cdk1 and phosphorylation of p27

**DOI:** 10.1038/s41598-019-54917-1

**Published:** 2019-12-10

**Authors:** Lucía García-Gutiérrez, Gabriel Bretones, Ester Molina, Ignacio Arechaga, Catherine Symonds, Juan C. Acosta, Rosa Blanco, Adrián Fernández, Leticia Alonso, Piotr Sicinski, Mariano Barbacid, David Santamaría, Javier León

**Affiliations:** 1 Instituto de Biomedicina y Biotecnología de Cantabria (IBBTEC), Universidad de Cantabria-CSIC, and Departmento de Biología Molecular, Universidad de Cantabria, Santander, Spain; 2 Edinburgh Cancer Research UK Centre, Institute of Genetics and Molecular Medicine, University of Edinburgh, Edinburgh, UK; 3 Experimental Oncology, Molecular Oncology Programme, Centro Nacional de Investigaciones Oncológicas (CNIO), Madrid, Spain; 4 Department of Cancer Biology, Dana-Farber Cancer Institute, Boston, USA; 5 University of Bordeaux, INSERM U1218, ACTION Laboratory, IECB, Pessac, France; 6 Present Address: Systems Biology Ireland, University College Dublin, Belfield, Dublin Ireland; 7 Present Address: Departamento de Bioquímica y Biología Molecular, Instituto Universitario de Oncología-IUOPA, Universidad de Oviedo, 33006 Oviedo, Spain; 8 Present Address: Global Oncology Franchise, EMD Serono, Rockland, Massachusetts USA

**Keywords:** Cancer, Cell biology, Molecular biology

## Abstract

Cell cycle stimulation is a major transforming mechanism of Myc oncoprotein. This is achieved through at least three concomitant mechanisms: upregulation of cyclins and Cdks, downregulation of the Cdk inhibitors p15 and p21 and the degradation of p27. The Myc-p27 antagonism has been shown to be relevant in human cancer. To be degraded, p27 must be phosphorylated at Thr-187 to be recognized by Skp2, a component of the ubiquitination complex. We previously described that Myc induces Skp2 expression. Here we show that not only Cdk2 but Cdk1 phosphorylates p27 at the Thr-187. Moreover, Myc induced p27 degradation in murine fibroblasts through Cdk1 activation, which was achieved by Myc-dependent cyclin A and B induction. In the absence of Cdk2, p27 phosphorylation at Thr-187 was mainly carried out by cyclin A2-Cdk1 and cyclin B1-Cdk1. We also show that Cdk1 inhibition was enough for the synthetic lethal interaction with Myc. This result is relevant because Cdk1 is the only Cdk strictly required for cell cycle and the reported synthetic lethal interaction between Cdk1 and Myc.

## Introduction

Progression through the cell cycle is controlled by a highly conserved family of serine/threonine protein kinases. These kinases are heterodimers that consist of a catalytic subunit, the cyclin-dependent protein kinase (Cdk) and a regulatory subunit, the cyclin, which is required for the Cdk to become active. Although Cdks and cyclins are large protein families, Cdk1, 2, 4 and 6 and A, B, E, D-type cyclins are identified as the major regulators of the cell cycle^1^. Systematic knockout of *Cdk* loci in the mouse germline has shown that Cdk2^2,3^, Cdk4^4,5^ and Cdk6^6^ are not essential for cell cycle progression of most cell types, although loss of each of these Cdks results in particular developmental defects. Moreover, concomitant loss of the genes of interphase Cdks does not result in a general disturbance of the cycle in most cell types, being Cdk1 alone sufficient and essential to drive the entire cycle^1,7^.

Myc (also called c-Myc) is an oncogenic transcription factor that belongs to the helix-loop-helix/leucine zipper family of proteins. Myc-mediated transcriptional activation depends on its interaction with Max, another helix-loop-helix transcription factor. Myc-Max heterodimers bind to DNA sequences known as E-boxes within the regulatory regions of their target genes and recruit transcriptional coactivators. Nevertheless, Myc has also the ability to repress gene transcription through less known mechanisms (for reviews see^8–10^). Myc is found deregulated in nearly half of human solid tumors and leukemia, and appears frequently associated with tumor progression^11–13^.

Induction of cell proliferation by promoting G_1_ to S-phase transition during cell cycle progression is one of Myc’s best characterized functions, a feature linked to its pro‐oncogenic activity. Indeed, enforced Myc expression in quiescent cells is sufficient to mediate cell cycle entry. At least three major mechanisms account for this: (i) the transcriptional activation of genes required for cell cycle progression, including a number of cyclins (D2, A, E); (ii) the repression of *CDKN2B* (p15) and *CDKN1A* (p21) genes and (iii) the degradation of CDKN1B/p27^KIP1^ (p27 here after) cell cycle inhibitor (reviewed in^14^).

The well-established Myc-p27 antagonism is one of the major mechanisms of Myc-mediated tumorigenic function. p27 is a Cdk inhibitor found downregulated in proliferating cells and in many tumors. Cyclin E-Cdk2 is considered p27’s primary target^15,16^, although other targets rather than Cdk2 have been proposed^17^. The ability of Myc to overcome the p27-mediated proliferative arrest has been demonstrated not only in cell culture^18,19^, but also in animal carcinogenesis models^20^. This antagonistic effect of Myc on p27 is mediated through several concomitant mechanisms: (i) Myc induces cyclin D2 and Cdk4, which sequester p27 allowing cyclin E-Cdk2 activation^21,22^; (ii) Myc induces expression of Cullin 1 (Cul1)^23^ and Cks1^24^, both components of the SCF^SKP2^ complex and (iii) we showed that Skp2, the p27-recognizing subunit of the SCF^SKP2^ ubiquitin ligase complex is a Myc target gene^25^. Moreover, Skp2 has been considered to have oncogenic potential and is found overexpressed in many human tumors^26,27^.

Previous studies indicated that p27 must be phosphorylated at Thr-187 to be recognized by the SCF^SKP2^ ubiquitin ligase complex, and thus being efficiently ubiquitinated and targeted for proteasome-mediated degradation^28,29^. In this work, we studied the mechanism of Myc-mediated phosphorylation of p27 independently of Cdk2 activity. Through genetic analysis based on loss of function of Cdk1 and Cdk2 along with conditional Myc expression, we show here the pivotal role of Cdk1 on p27 phosphorylation and its potential relevance for Cdk1-based synthetic lethal approaches to control Myc in cancer.

## Results

### Myc induces phosphorylation of p27 mediated by Cdk1 and Cdk2 in human leukemia cells

**Figure 1:**
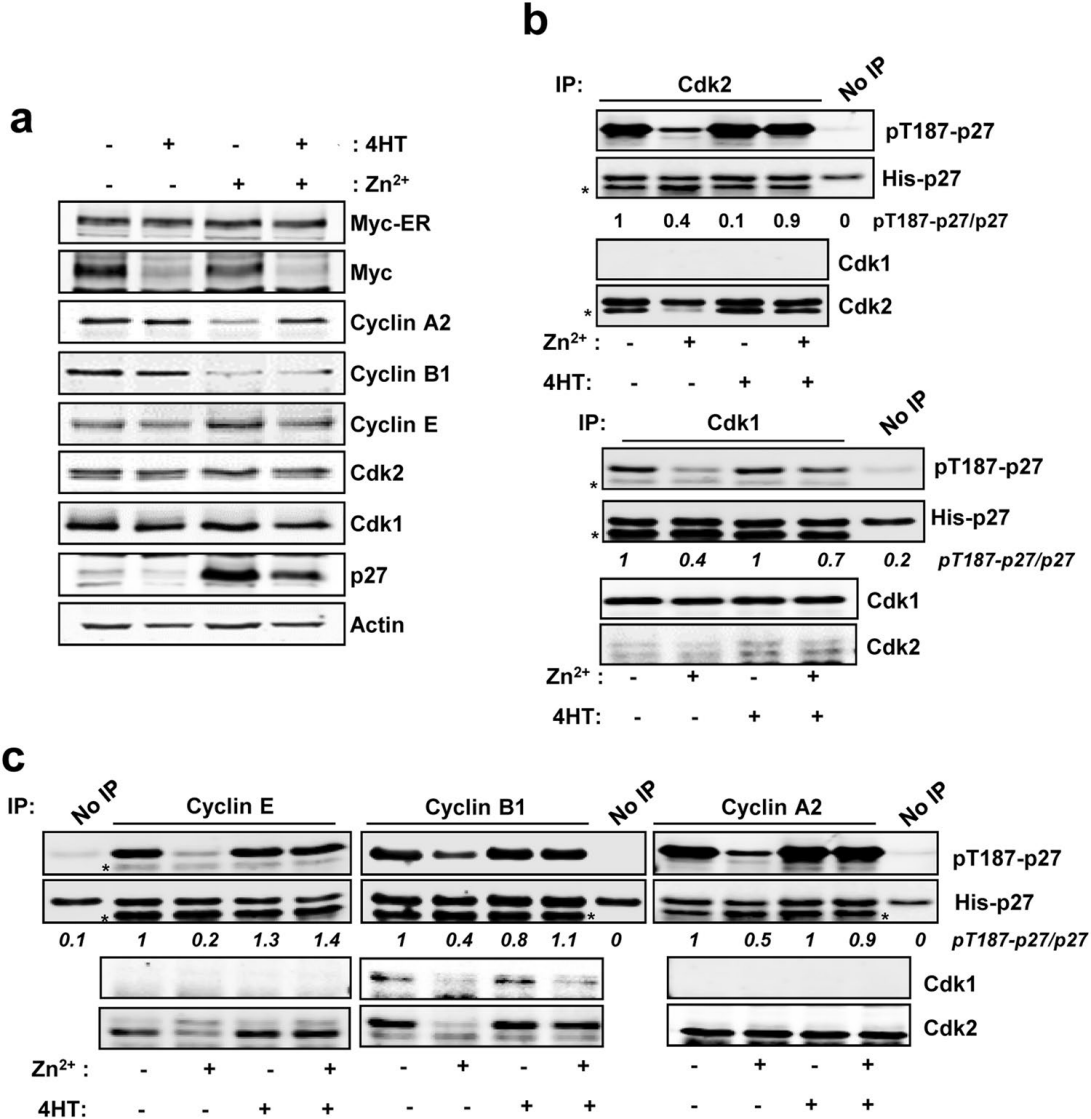
Myc-mediated induction of p27 phosphorylation in leukemia cells. (**a**) Kp27MER cells were treated with 50 µM Zn^2+^ and 200 nM 4HT for 24 hours as indicated to induce ectopic p27 expression and Myc-ER activation. Myc, Myc-ER, cyclins A2, B1 and E, Cdk1, Cdk2 and p27 levels are shown. Actin levels were used as loading control. (**b**) Immunoprecipitated Cdk2 (upper panel) and Cdk1 (lower panel) complexes from Kp27MER cells treated with Zn^2+^ and 4HT for 12 hours as indicated and subjected to *in vitro* kinase assays with His-p27 as substrate. Levels of Cdk1 and Cdk2 in immunoprecipitates are also shown. **(c)** Immunoprecipitated cyclin B1, cyclin A2, cyclin E complexes from Kp27MER treated with Zn^2+^ and 4HT for 12 hours as indicated and subjected to *in vitro* kinase assays using His-p27 as substrate. Levels of Cdk1 and Cdk2 present in cyclin A2, cyclin B1 and cyclin E are also shown. Asterix (*) in kinase assays shows light chain of the antibody from immunoprecipitation Signal densitometry quantification of the kinase assays are shown below each lane. Kinase buffer with His-p27 was used as negative control (No IP).

Previous results in our laboratory in a human myeloid leukemia cell line K562 have shown that Myc´s ability to promote cell cycle progression depends on the reduction of p27 (p27^KIP1^, CDKN1B) protein levels^19^. We used a K562 derivative cell line, called Kp27MER, which contains a Zn^2+^- inducible p27 construct and the chimeric protein Myc-ER, which is constitutively expressed but only active in presence of 4-hydroxi-tamoxifen (4HT). In this model, induction of p27 lead to arrested proliferation, while Myc-ER activation by 4HT induced p27 phosphorylation at the Thr-187 and partially rescued the proliferative state^19,25^. We first confirmed that concomitant induction of p27 and Myc-ER activation resulted in decreased p27 levels, as expected (Fig. 1a). p27 induction decreased cyclin A2 expression and this was counteracted by concomitant activation of Myc-ER, consistently with the proliferation arrest exerted by p27 and its partial rescue by Myc. These changes were not observed in parental K562 cells (Supplementary Fig. S1a). Downregulation of endogenous Myc upon 4HT addition is a marker of the Myc-ER activation (Fig. 1a). Expression of cyclins B and E, and Cdk1 and Cdk2 upon those conditions are also shown and did not show major changes (Fig. 1a). The decrease of p27 in Kp27MER is linked to Myc-mediated phosphorylation of p27^25^. Thus, we investigated whether Myc-mediated p27 phosphorylation was specific for cyclin E-Cdk2 complexes as previously reported^30,31^ or if other cyclin-cdk complexes could be involved due to their redundancy.

Upon high p27 levels (i.e., by addition of Zn^2+^ in our model), Cdk1 and Cdk2 complexes are inhibited as expected and demonstrated by *in vitro* kinase assays using His-p27 as substrate (Fig. 1b). However, activation of Myc-ER under these conditions rescued the impaired kinase activity over p27 of, not only Cdk2, but also Cdk1 (Fig. 1b). To investigate which cyclin regulates Cdk1/2 kinase activity upon Myc-ER activation, cyclins A2, B1 and E were immunoprecipitated (Supplementary Fig. S1b) and subjected to *in vitro* kinase assays under the same conditions as before. The results showed that the kinase activity of the three immunocomplexes was low in cells expressing p27 (i.e., treated with Zn^2+^) but was dramatically enhanced upon Myc activation (i.e., with 4HT) (Fig. 1c). However, differences are found within the interaction affinities between certain cyclins and cdks. These results suggest that not only Cdk2 but also Cdk1 could phosphorylate p27 at the Thr-187 promoting its degradation (in agreement with the well-known redundancy of cyclin-cdk complexes) and that Myc regulates such activity.

### Myc stimulates proliferation and p27 degradation in cells lacking Cdk2 or cyclin E-associated kinase activity

**Figure 2:**
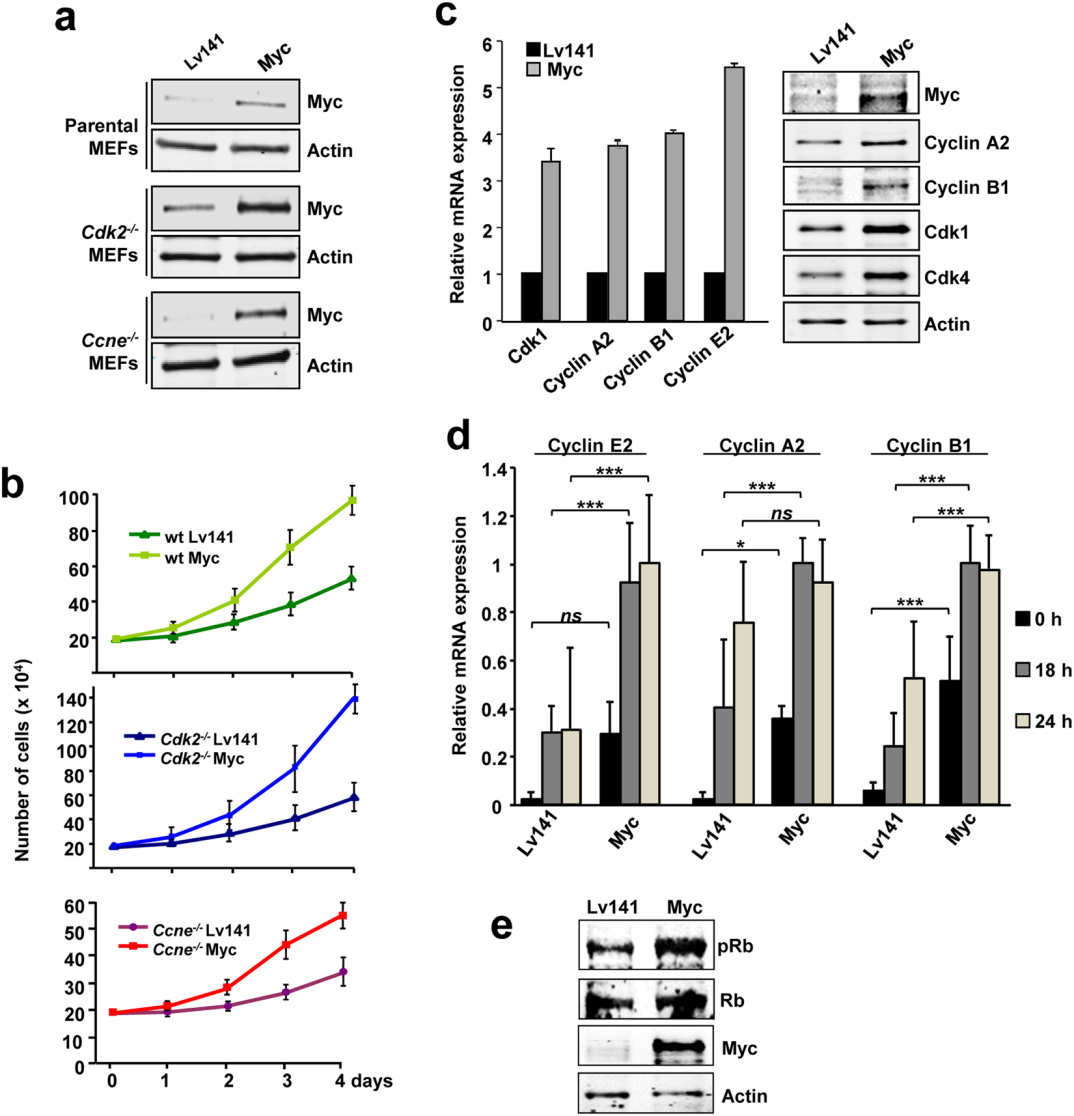
Proliferation rates of Myc-transduced *Cdk2*^−/−^ and *Ccne*^−/−^ MEFs. (**a**) Myc transduction of parental, C*dk2*^−/−^ and *Ccne*^−/−^ MEFs analyzed by western blot. Actin levels were used as loading control. (**b**) Proliferation curves of wild-type (wt), *Cdk2*^−/−^ and *Ccne*^−/−^ MEFs stably transduced with a constitutive Myc vector compared with controls (Lv141). Data is shown as total number of cells per condition. Error bars represent ± SD of two independent experiments. (**c**) Left panel**:** mRNA expression of *Cdk1*, *Ccna2*, *Ccnb1* and *Ccne2* genes analyzed by RT-PCR and normalized to *β-actin* levels. Error bars represent ± SD of at least two independent experiments. Right panel: Protein expression of Myc, cyclin A2, cyclin B1, Cdk1 and Cdk4 analysed by western blot. Actin levels were used as loading control. (**d**) mRNA expression of *Ccne2*, *Ccna2* and *Ccnb1* of *Cdk2*^−/−^ Lv141 MEFs and *Cdk2*^−/−^ Myc MEFs in either serum-starved (0 h) or serum-stimulated (18–24 h) conditions determined by RT-PCR and normalized against the maximum expression of each cyclin within both cell lines. Error bars represent ± SD of five independent experiments. Statistical analysis: 2 way-ANOVA.*P < 0.033 and ***P < 0.001. (**e**) Phosphorylation of Rb protein analysed by western blot in *Cdk2*^−/−^ Lv141 and *Cdk2*^−/−^ Myc MEFs using a phospho-specific antibody for pRb.

It is known that p27 levels within the cell are mainly controlled by cyclin E-Cdk2, and that Myc mainly relies on cyclin E-Cdk2 activity to phosphorylate p27. Thus, and in agreement with the fact that Cdk1 could substitute Cdk2 in such task, we set out a series of experiments to elucidate the potential role of Cdk1 on Myc-dependent stimulation of cell cycle progression independently of Cdk2 activity. First, we compared the effect of Myc enforced expression on the proliferation of wild-type MEFs and MEFs lacking either cyclin E or Cdk2 expression (wild type, *Cdk2*^−/−^ and *Ccne*^−/−^ MEFs respectively). Myc enforced expression was achieved by lentiviral transduction and confirmed by western blot (Fig. 2a). Comparison between the proliferation rates of *Cdk2*^−/−^ cells and *Ccne*^−/−^ cells with that of their overexpressing Myc counterparts showed that exogenous Myc overexpression increases proliferation rates of *Cdk2*^−/−^ as well as that of *Ccne*^−/−^ MEFs (Fig. 2b).

To explore the mechanisms through which Myc increases cell proliferation independently of cyclin E-Cdk2 complexes, we first studied the expression of cyclins and Cdks involved in cell cycle progression in *Cdk2*^−/−^ MEFs. The results showed that in Myc-overexpressing cells, Cdk1, Cdk4, cyclin A2, cyclin B1 and cyclin E2 expression were higher than in control Lv141 cells at both, mRNA and protein levels (Fig. 2c). Cell starvation followed by serum stimulation of *Cdk2*^−/−^ MEFs revealed that upon Myc overexpression, the induction of cyclins at the mRNA level was accelerated in time when compared with control cells infected with the empty viral vector (Lv141) and the cyclin expression levels achieved were higher (Fig. 2d). Indeed, basal mRNA expression levels were already higher after starvation in Myc-overexpressing cells compared to controls (Fig. 2d). Consistently, we found that retinoblastoma was hyperphosphorylated in Myc-overexpressing cells (Fig. 2e). In summary, we showed that Myc overexpression induced proliferation in cells deficient in either cyclin E or Cdk2 and this was accompanied with an increase in cyclin A2 and B1 expression, as described in other models^32–35^.

**Figure 3:**
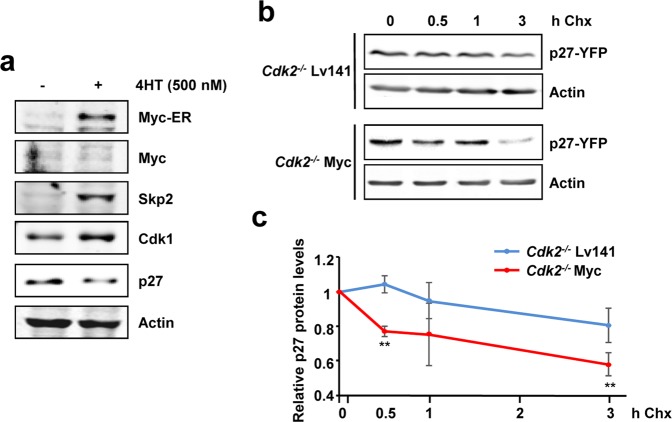
Myc-mediated degradation of p27. (**a**) Protein levels of Myc, Skp2, Cdk1 and p27 of *Cdk2*^−/−^ MER MEFs grown until confluence and treated with 500 nM of 4HT for 18 hours (overnight). Actin levels were used as loading control. (**b**) Protein stability of p27 in *Cdk2*^−/−^ Lv141 and *Cdk2*^−/−^ Myc MEFs transfected with a p27-YFP construct measured by western blot. Levels of p27-YFP were detected after 0, 0.5, 1 and 3 hours of cycloheximide treatment (30 µg/mL). Actin levels were used as loading control. (**c**) Densitometric quantification of p27 protein levels after cycloheximide treatment normalized to actin levels. Error bars represent ± SD of three independent experiments. Statistical analysis: T.test: *p < 0.1; **p < 0.05.

The main degradation pathway of p27 has been described to rely on Cdk2 kinase activity. Due to the opposite correlation between p27 and Myc in proliferating and tumor cells, we analyzed whether in the absence of Cdk2 kinase activity Myc was able to reduce p27 protein levels. First, p27 levels were analyzed in *Cdk2*^−/−^ MEFs expressing the chimeric protein Myc-ER (termed *Cdk2*^−/−^ MER MEFs). Cells were grown until confluence to enforce p27 accumulation and then treated with 4HT to activate Myc-ER. Activation of Myc-ER was accompanied by increased levels of Cdk1 and Skp2, while p27 protein levels were reduced (Fig. 3a). The increase in Skp2 levels is consistent with the reduction of p27 and Myc activation, as Skp2 is a direct Myc target gene responsible of the recognition of p27 to be further degraded^25^. Thus, to investigate the effect of Myc on p27 stability in the absence of Cdk2, *Cdk2*^−/−^ Myc MEFs and their corresponding control *Cdk2*^−/−^ Lv141 MEFs were transfected with a p27-YFP construct and treated with cycloheximide, a protein synthesis inhibitor. After different periods of cycloheximide treatment, p27 protein levels were analyzed by western blot (Fig. 3b) and quantified (Fig. 3C). Myc protein levels (a well-known short half-life protein) are shown as a technical control of the efficacy of the cycloheximide treatment (Supplementary Fig. S2). The results showed that p27 protein levels decreased faster in cells overexpressing Myc than in control cells, suggesting that Myc affects p27 stability in the absence of Cdk2. In summary we have shown that Myc was able to promote proliferation and p27 degradation in *Cdk2*^−/−^ MEFs.

Myc-mediated degradation of p27 can be explained by Myc induction of Skp2^25^, an effect that we have confirmed in MEFs (Fig. 3b). However, to be recognized by Skp2, p27 must be phosphorylated at Thr-187, a phosphorylation that has been reported to be mediated by cyclin E/A-Cdk2^30,31^. We have shown here that Cdk1 could phosphorylate p27 in human leukemia cells (Fig. 1b). Indeed, previous reports demonstrated that *Cdk2*^−/−^ mice showed phosphorylated p27 at the Thr-187^3^ and that cyclin B-Cdk1 could phosphorylate p27 *in vitro* but in a lesser extent when compared with Cdk2 complexes^36^. Thus, we hypothesized that in the absence of Cdk2, Myc would induce p27 phosphorylation through Cdk1 activation.

**Figure 4:**
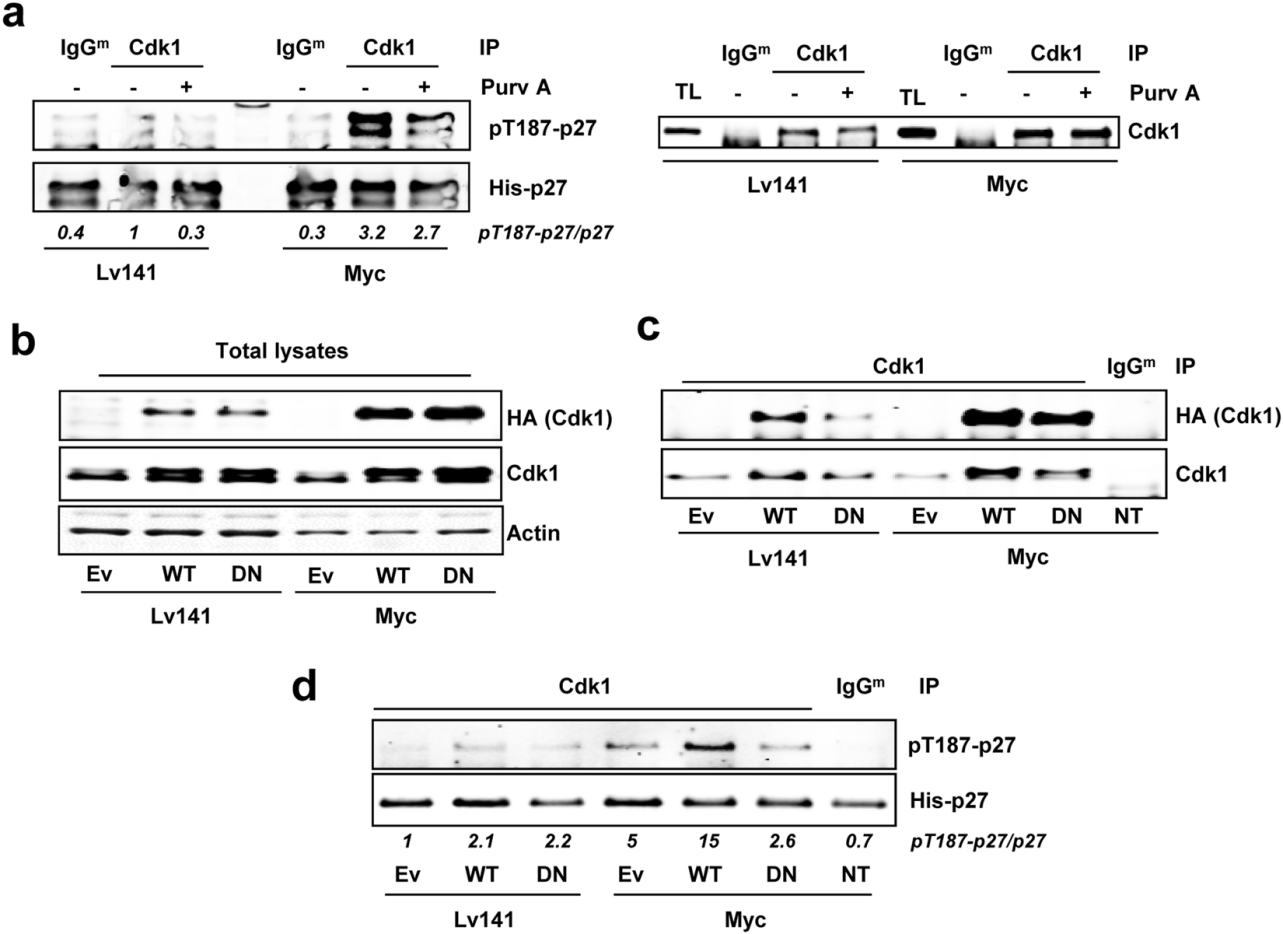
Cdk1 mediated phosphorylation of p27 at the Thr-187. (**a**) Left panel: *In vitro* kinase assay of Cdk1 immunoprecipitated from *Cdk2*^−/−^ Lv141 and *Cdk2*^−/−^ Myc MEFs using His-p27 as substrate. Phosphorylation levels of p27 at the Thr-187 were detected using a phospho-specific antibody. Right panel: Levels of the immunoprecipitated Cdk1 assayed in the experiment of the left panel measured by western blot. (**b**) Total lysates from *Cdk2*^−/−^ Lv141 and *Cdk2*^−/−^ Myc MEFs transfected with empty vector (Ev) HA-Cdk1-wt (WT), HA-Cdk1-DN (DN) or non-transfected (NT). Total Cdk1 and exogenous Cdk1 are shown (anti-Cdk1 and anti-HA antibodies respectively). Actin levels were used as loading control. (**c**) Total Cdk1 immunoprecipitated from protein extracts showed in the left panel and immunoprecipitated exogenous Cdk1 levels measured with anti-HA. (**d**) Kinase activity of Cdk1 wild-type (WT) and Cdk1DN (DN) on Thr-187 of p27. Immunocomplexes were treated with 10 µM purvalanol A (Purv A) or vehicle (DMSO) in the kinase assays when indicated. Signal densitometry quantification of the kinase assays are shown below each lane. Unspecific IgG^m^ was used as negative control for the specificity of the antibody used for immunoprecipitation.

To confirm this hypothesis, we first explored p27 phosphorylation by Cdk1 at the Thr-187 in *Cdk2*^−/−^ MEFs. Thus, Cdk1 immunocomplexes were prepared from *Cdk2*^−/−^ Myc MEFs and *Cdk2*^−/−^ Lv141 MEFs and subjected to *in vitro* kinase assays to test their activity on p27 at the Thr-187 and whether Myc increases it. The amount of immunoprecipitated Cdk1 was assayed by western blot (Fig. 4a right panel). As shown in Fig. 4a left panel, Cdk1 immunocomplexes efficiently phosphorylated p27. Cdk1-mediated phosphorylation of p27 resulted higher in immunocomplexes obtained from *Cdk2*^−/−^ Myc cells when compared to control cells. Besides, treatment of the immunocomplexes with the Cdk1 inhibitor purvalanol A led to a decrease in p27 phosphorylation levels compared to untreated ones.

To verify Cdk1-mediated p27 phosphorylation, we transfected *Cdk2*^−/−^ Myc MEFs and *Cdk2*^−/−^ Lv141 MEFs with an inactive mutant of Cdk1 lacking kinase activity (Cdk1^D146N^, Cdk1^DN^ herein after) and we performed *in vitro* kinase assays. The empty vector (EV) and wild-type Cdk1 were used as controls. The transfected wild-type Cdk1 and Cdk1^DN^ were tagged with an HA epitope, which served to detect the exogenous Cdk1 (Fig. 4b). Due to the poor efficiency of the anti-HA antibody to immunoprecipitate our HA-tagged Cdk1 constructs, Cdk1 immunocomplexes were prepared with an anti-Cdk1 antibody followed by *in vitro* p27 phosphorylation assay. The exogenous immunoprecipitated Cdk1 was detected by western blot with anti-HA antibody (Fig. 4c). The kinase assays showed that overexpression of Cdk1^wt^ in *Cdk2*^−/−^ Myc MEFs led to higher phospho-p27 levels while the Cdk1^DN^ form did not show this effect when compared with the empty vector. Furthermore, overexpression of either wild-type Cdk1 or Cdk1^DN^ showed no major differences in p27 phosphorylation compared with the empty vector in *Cdk2*^−/−^ Lv141 MEFs (Fig. 4d). This suggests that the limiting factor in p27 phosphorylation is not Cdk1 but cyclin levels, as higher Cdk1 protein levels do not have any effect unless Myc is overexpressed.

**Figure 5:**
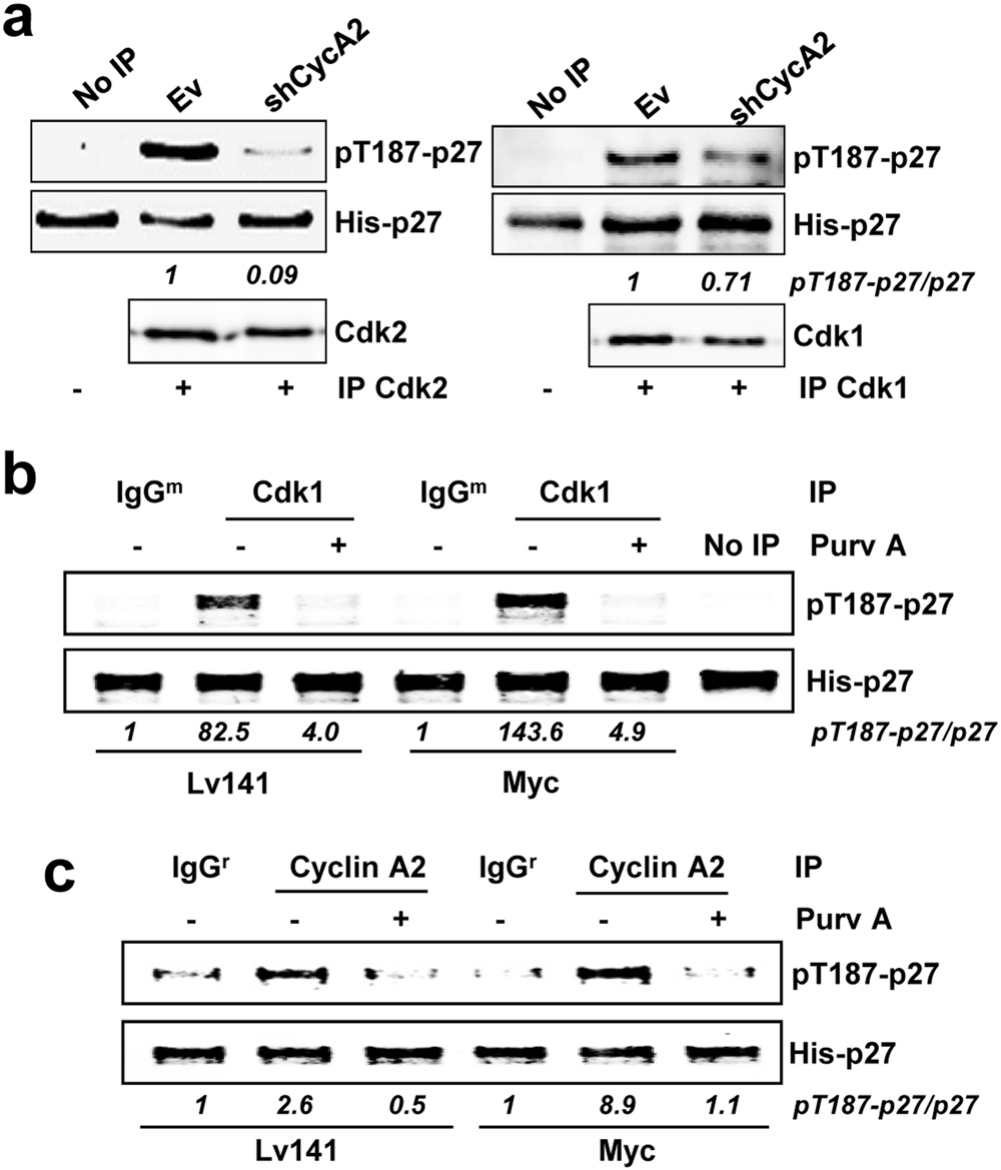
p27 is phosphorylated at the Thr-187 in *Ccne*^−/−^ MEFs. (**a**) *In vitro* kinase assays of Cdk2 and Cdk1 from *Ccne*^−/−^ MEFs using His-p27 as substrate. Immunoprecipitated Cdk2 and Cdk1 are shown. (**b**) Cdk1 from *Ccne*^−/−^ Lv141 or *Ccne*^−/−^ Myc MEFs was immunoprecipitated and assayed *in vitro* over p27. (**c**) Cyclin A2 from *Ccne*^−/−^ Lv141 or *Ccne*^−/−^ Myc MEFs was immunoprecipitated and assayed *in vitro* over p27. Immunocomplexes were treated with 10 µM purvalanol A (Purv A) or vehicle (DMSO) in the kinase assays when indicated. Signal densitometry quantification of the kinase assays are shown below each lane. Kinase buffer with His-p27 was used as negative control (No IP) and unspecific IgG was used as negative control for the specificity of the antibody used for immunoprecipitation.

The above results demonstrated that Myc stimulates the phosphorylation of p27 mediated by Cdk1. Next, we investigated the cyclin partner of Cdk1 in this function. For this purpose, we used cells deficient in cyclin E1/E2. Both Cdk1 and Cdk2 were able to phosphorylate p27 *in vitro* in the absence of cyclin E (Fig. 5a). We silenced cyclin A2 expression in *Ccne*^−/−^ MEFs by shRNA lentiviral transduction (Supplementary Fig. S3a). Both Cdk1 and Cdk2 immunocomplexes showed reduced *in vitro* kinase activity on p27 (Fig. 5a). These results show that Cdk1-mediated phosphorylation of p27 is regulated, in part, by cyclin A2, a function unreported so far. However, Cdk1 activity was affected in a lesser extent by cyclin A2 downregulation than that of Cdk2, suggesting that cyclin B could be also regulating Cdk1-mediated p27 phosphorylation in these cells, consistently with results previously reported^37^. We also tested whether Myc increased p27 phosphorylation in this model. *Ccne*^−/−^ MEFs were transduced with a Myc-IRES-GFP construct or the empty vector (Lv141) and sorted by GFP expression. Cdk1 or cyclin A2 were immunoprecipitated (Supplementary Fig. S3B) and the *in vitro* kinase assays showed that Myc overexpression increased Cdk1-mediated p27 phosphorylation (Fig. 5b), as well as cyclin A2-associated kinase activity on p27 (Fig. 5c). Total levels of cyclin A2 and Cdk1 were assessed by western blot (Supplementary Fig. S3a). Purvalanol A treatment abolished p27 phosphorylation in all cases. Altogether the results showed that both Cdk1 and Cdk2 were able to phosphorylate p27 at the Thr-187 in a cyclin E independent manner, suggesting that, in addition to cyclin A-Cdk2, cyclin A-Cdk1 and/or cyclin B-Cdk1 might be responsible for p27 phosphorylation.

**Figure 6:**
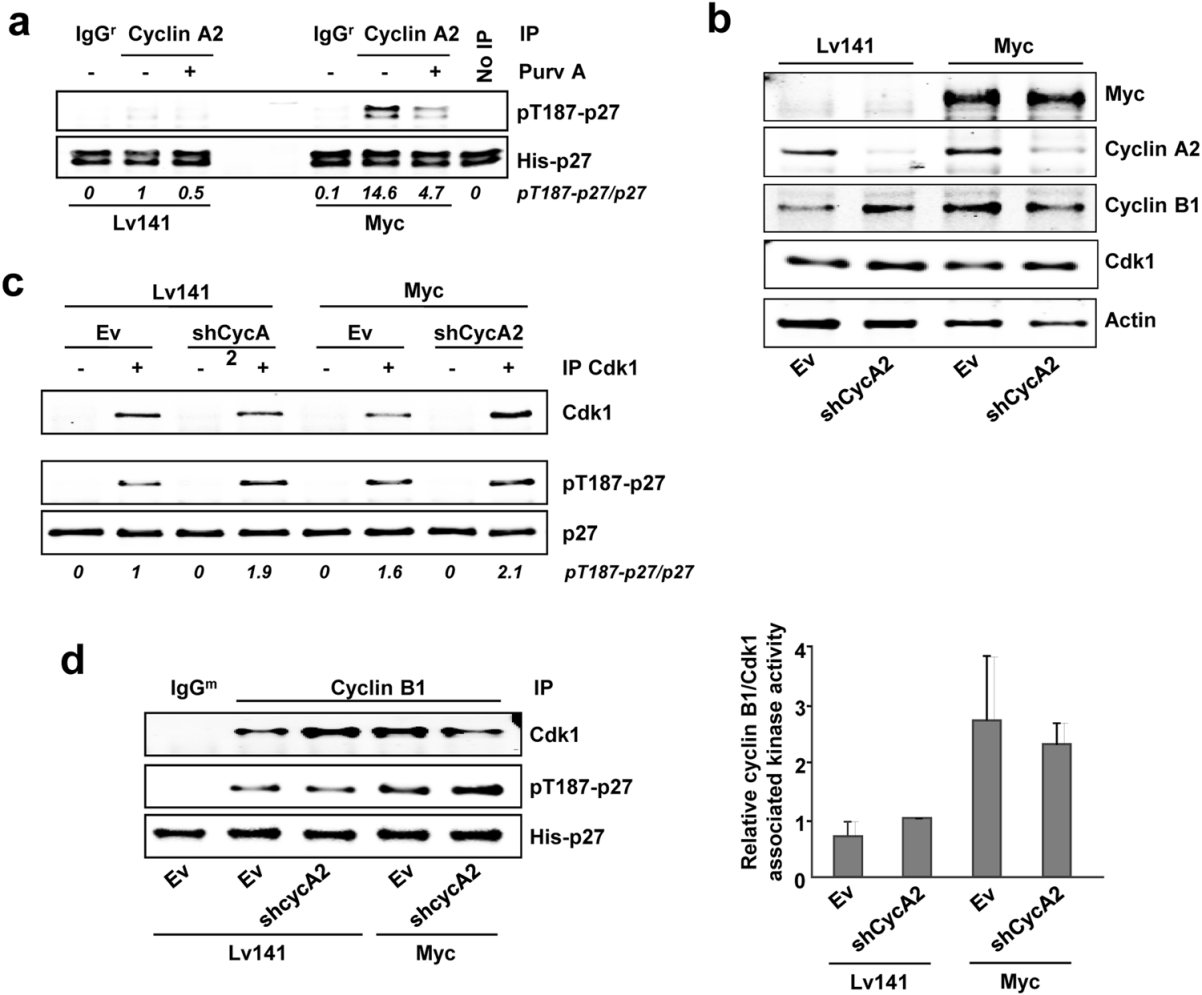
Cyclin A2 is not essential for Cdk1 to phosphorylate p27 *in vitro*. (**a**) *In vitro* kinase assay of cyclin A2 complexes from *Cdk2*^−/−^ Lv141 and *Cdk2*^−/−^ Myc MEFs using His-p27 as substrate. (**b**) Total lysates of knocked down cyclin A2 *Cdk2*^−/−^ Lv141 and *Cdk2*^−/−^ Myc MEFs assayed by western blot. Cyclin A2, cyclin B1, Cdk1 and Myc levels are shown. Actin levels were used as loading control. (**c**) *In vitro* kinase assay of Cdk1 complexes from *Cdk2*^−/−^ Lv141 and *Cdk2*^−/−^ Myc MEFs with knocked down cyclin A2 expression. The amount of immunoprecipitated Cdk1 is shown. (**d**) Left panel**:** *In vitro* kinase assay of cyclin B1 complexes from *Cdk2*^−/−^ Lv141 and *Cdk2*^−/−^ Myc MEFs with knocked down cyclin A2 expression. Right panel: Densitometry quantification of the relative cyclin B1 kinase activity. Error bars represent ± SD of the quantification of two independent experiments. Immunocomplexes were treated with 10 µM purvalanol A (Purv A) or vehicle (DMSO) in the kinase assays when indicated. Kinase buffer with His-p27 was used as negative control (No IP) and unspecific IgG was used as negative control for the specificity of the antibody used for immunoprecipitation.

Next, we tested the potential role of cyclin A2 as Cdk1 partner in Lv141 and Myc-overexpressing *Cdk2*^−/−^ MEFs. Kinase assays of cyclin A2 complexes from *Cdk2*^−/−^ MEFs led to p27 phosphorylation and was increased by Myc. Besides, treatment of the complexes with purvalanol A decreased p27 phosphorylation (Fig. 6a). Furthermore, we analyzed the requirement of cyclin A2 in this function. Knocking down cyclin A2 in *Cdk2*^−/−^ Lv141 and *Cdk2*^−/−^ Myc MEFs was achieved by shRNA lentiviral transduction (Fig. 6b). In order to test the efficiency of cyclin A2 knock down, we performed *in vitro* kinase assays of cyclin A2 upon these conditions and we found that downregulation of cyclin A expression lead to a reduction in p27 phosphorylation as expected (Supplementary Fig. S4). Furthermore, kinase assays showed that Myc overexpression resulted in increased Cdk1-mediated p27 phosphorylation, but cyclin A2 depletion did not affect it (Fig. 6c). These results showed that the increased kinase activity of Cdk1 did not depend only on the upregulation of cyclin A2 by Myc. Thus, we explored cyclin B1 as another potential regulator of Cdk1-mediated p27 phosphorylation induced by Myc. Immunoprecipitated cyclin B1 complexes from *Cdk2*^−/−^ Lv141 MEFs were able to phosphorylate p27 *in vitro*, as expected, while Myc increased cyclin B1-associated kinase activity on p27. Moreover, downregulation of cyclin A2 levels did not affect it (Fig. 6d). Interaction of Cdk1 with cyclins A2 and B1 in these cells is shown in Supplementary Fig. S5.

### Myc induces proliferation and Cdk1-mediated p27 phosphorylation in triple knock out MEFs

As Cdks show redundant functions within the cells, we decided to use a triple knock out MEF cell line lacking *Cdk2*, *Cdk4* and *Cdk6* functional genes (*Cdk2*^−/−^; *Cdk4*^−/−^; *Cdk6*^−/−^ MEFs, termed TKO MEFs herein after). This model would help to verify that Cdk1 was also responsible for the phosphorylation of p27 upon Myc expression. TKO MEFs were transduced with a Myc-IRES-GFP construct and GFP expressing cells were sorted. The resulting cell line was termed TKO-Myc MEFs.

**Figure 7:**
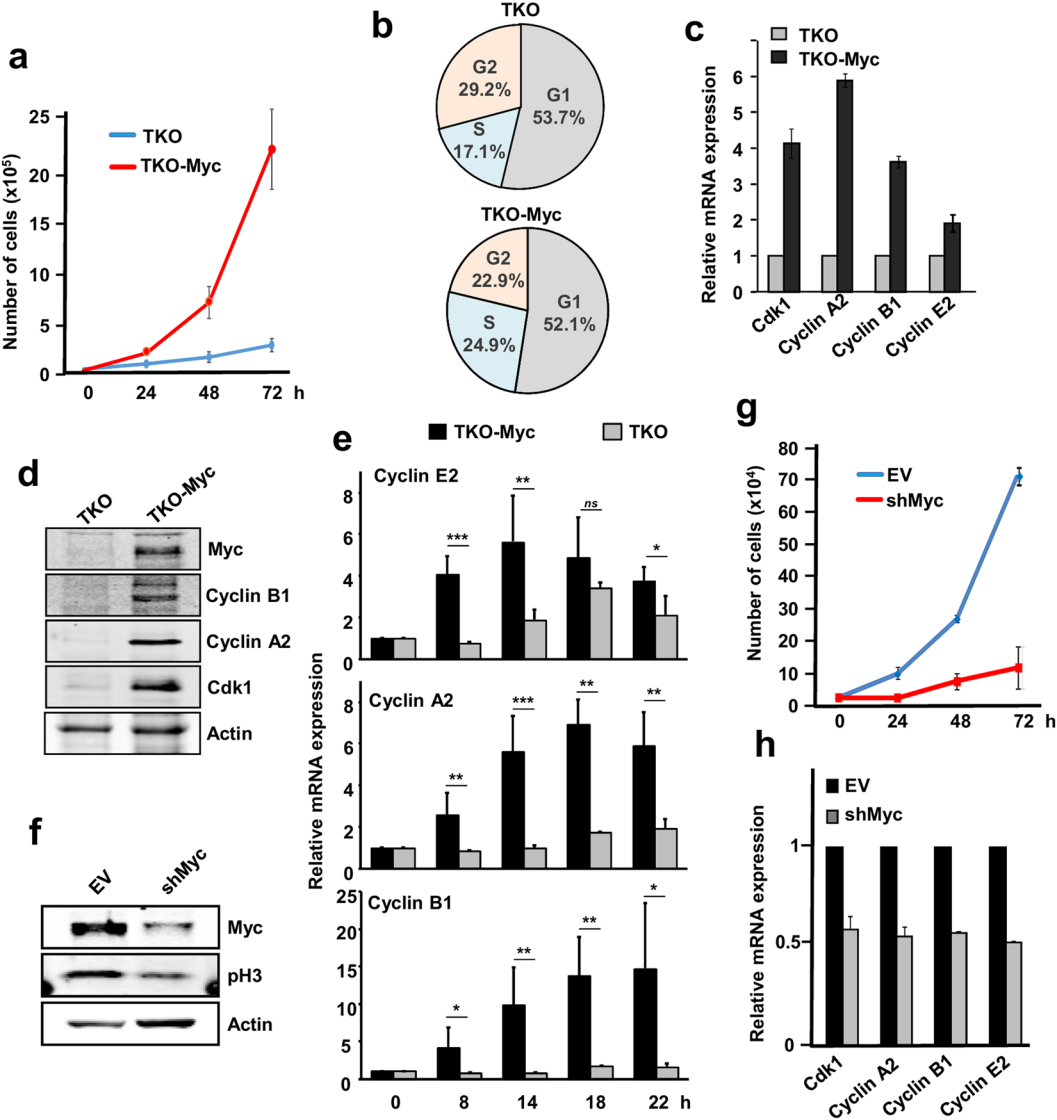
Myc induces proliferation and the expression of cell cycle-related genes in *Cdk2*^−/−^; *Cdk4*^−/−^ and *Cdk6*^−/−^ (TKO) MEFs. (**a**) Proliferation curves of TKO and TKO-Myc MEFs. Error bars represent ± SD of three independent experiments. (**b**) Cell cycle distribution in TKO and TKO-Myc MEFs. Data represent the average of independent experiments with similar results. (**c**) mRNA expression of *Ccna2*, *Ccnb1*, *Ccne2* and *Cdk1* analyzed by RT-PCR of TKO MEFs and TKO-Myc MEFs. mRNA expression normalized against *β-actin* levels. Error bars represent ± SD of two independent experiments. (**d**) Cyclin B1, cyclin A2, Cdk1 and Myc protein levels analyzed by western blot in TKO and TKO-Myc MEFs. Actin levels are used as loading control. (**e**) mRNA expression of *Ccne2*, *Ccna2* and *Ccnb1* at the indicated periods of time after serum stimulation in serum-starved TKO and TKO-Myc MEFs determined by RT-PCR. mRNA expression normalized against *β-actin* levels. Error bars represent ± SD of three independent experiments. Statistical analysis: T.test: *p < 0.1; **p < 0.05; ***p < 0.01. (**f**) TKO-Myc MEFs with downregulated Myc expression by shRNA transduction for *MYC* gene. Myc and phospho-H3 levels are shown. Actin levels are used as loading control. (**g**) Proliferation curve of TKO-Myc MEFs compared with Myc knocked down TKO Myc MEFs by shRNA transduction. Error bars represent ± SD of two independent experiments. (**h**) mRNA expression of *Ccna2*, *Ccnb1*, *Ccne2* and *Cdk1* mRNA analyzed by RT-PCR of TKO-Myc MEFs and TKO-Myc transduced with shMyc lentivirus. mRNA expression normalized against *β-actin* levels. Error bars represent ± SD of two independent experiments.

First, comparison between TKO and TKO-Myc MEFs proliferation rates showed that Myc overexpressing cells grew much faster than parental cells (Fig. 7a), in agreement with an increase in the percentage of cells in S-phase (Fig. 7b; Supplementary Fig. S6a). Consistently, *Cdk1*, *Ccna2*, *Ccnb1* and *Ccne2* expression was elevated in cells overexpressing Myc at the mRNA (Fig. 7c) and protein level (Fig. 7d). Moreover, the expression of cyclins A2, B1 and E2 was not only higher in TKO-Myc than parental TKO MEFS but also induced earlier in time upon serum stimulation (Fig. 7e). This verifies the importance of Myc as a cell cycle regulator, even in cells whose cell cycle is only driven by Cdk1. In agreement, downregulation of exogenous Myc in TKO-Myc MEFs by shRNA transduction (Fig. 7f) lead to arrested cell proliferation (Fig. 7g) and reduced expression of *Cdk1*, *Ccna2*, *Ccnb1* and *Ccne2* (Fig. 7h).

**Figure 8:**
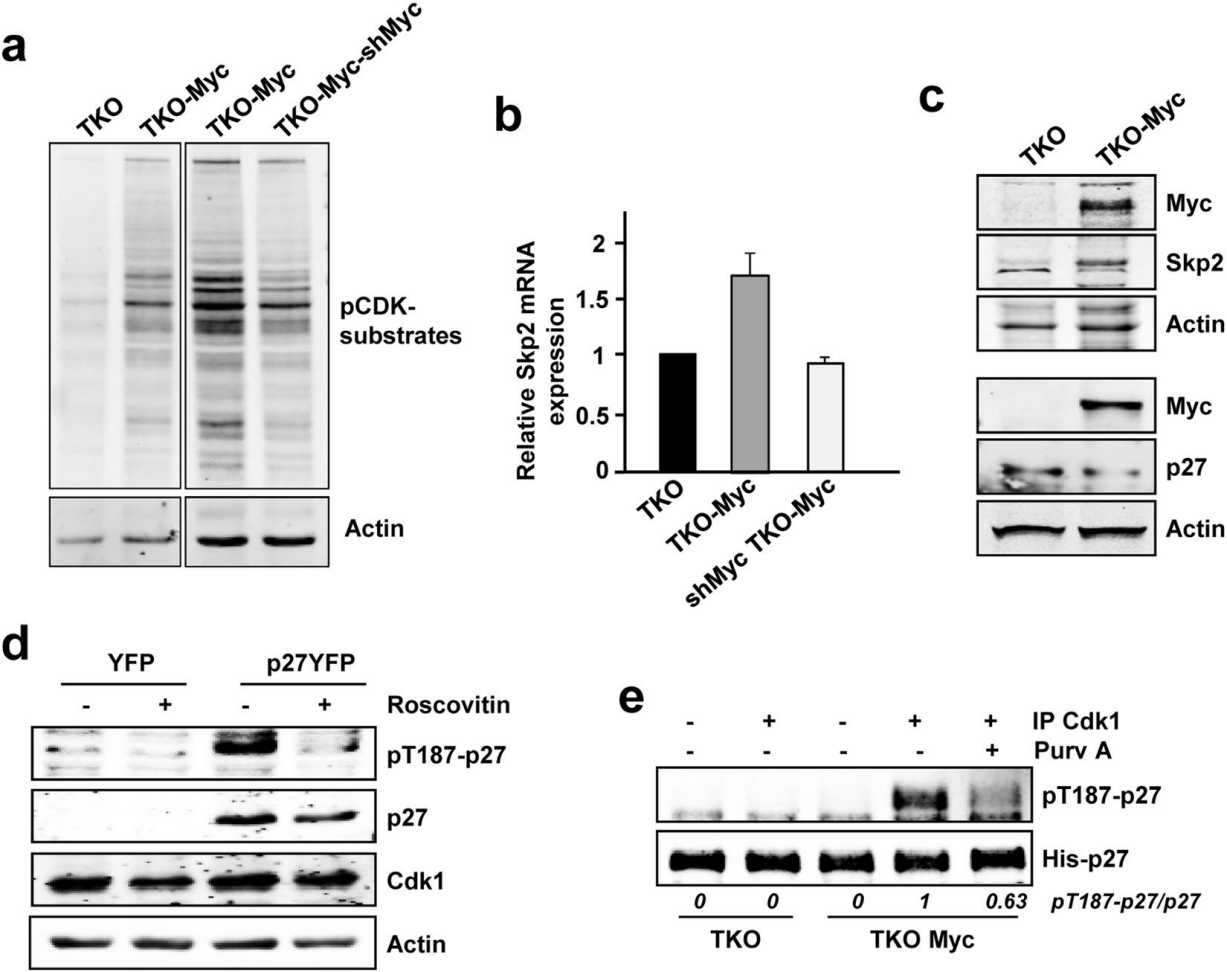
Myc induces p27 downregulation and phosphorylation in TKO MEFs. (**a**) Protein extracts from TKO, TKO-Myc and shMyc TKO-Myc MEFs showing total Cdk activity measured by western blot using a phospho-specific antibody for the consensus sequence recognized and phosphorylated by Cdks. Actin levels are used as loading control. (**b**) mRNA expression of *Skp2* in TKO, TKO-Myc and shMyc-TKO-Myc MEFs. mRNA expression is normalized to *β actin* levels. Error bars represent ± SD of two independent experiments. (**c**) Protein levels of Myc, p27 and Skp2 from TKO and TKO-Myc MEFs are shown. Myc and actin signals of the upper blot are the same than in Fig. 7d. (**d**) pT187-p27, total p27, Myc and Cdk1 levels analysed in protein extracts from YFP-p27 transfected TKO-Myc MEFs and treated with 10 µM roscovitine for 6 h. Actin levels were used as loading control. (**e**) *In vitro* kinase assays of Cdk1 immunocomplexes from TKO and TKO-Myc MEFs on His-p27 as substrate. Immunocomplexes were treated with 10 µM purvalanol A (Purv A) or vehicle (DMSO) in the kinase assays when indicated. Signal densitometry quantification of the kinase assays are shown below each lane. Unspecific IgG was used as negative control for the specificity of the antibody used for immunoprecipitation.

Since the only Cdk involved in cell cycle progression of TKO MEFs is Cdk1, we wondered whether Myc was able to induce Cdk1 kinase activity in these cells. We first investigated the presence of Cdk1-phosphorylated substrates using a phospho-specific antibody against the consensus amino acid sequence recognized and phosphorylated by Cdks. The results showed that Myc overexpression resulted in higher Cdk activity in TKO MEFs whereas downregulation of exogenous Myc levels in TKO-Myc MEFs lead to a decrease in total Cdk activity (Fig. 8a). Moreover, Myc overexpression in TKO MEFs induced *Skp2* levels when compared with control TKO cells in the same extent as in other models. In agreement, knocking down ectopic Myc expression lead to a decrease in *Skp2* levels (Fig. 8b), which is consistent with the results previously obtained using the *Cdk2*^−/−^ MEFs cell line. Besides, Skp2 and p27 levels show opposite expression profiles upon Myc overexpression (Fig. 8c).

**Figure 9:**
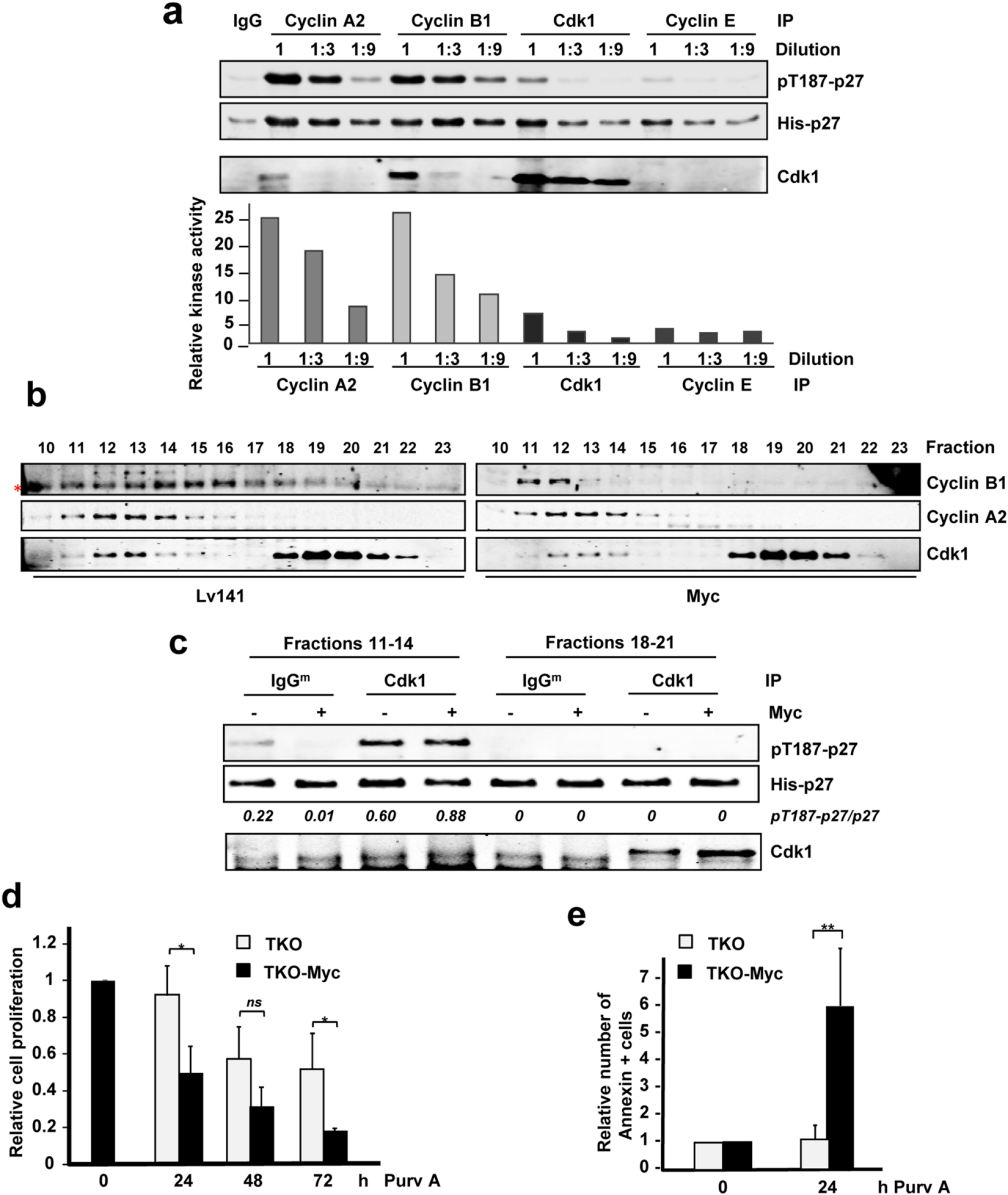
Cyclin A2 and cyclin B1 associated to Cdk1 phosphorylate p27 at the Thr-187 in MEFs lacking Cdk2. (**a**) *In vitro* kinase assays of cyclin A2, cyclin B1, cyclin E2 and Cdk1 complexes from TKO-Myc MEFs using His-p27 as substrate. The immunoprecipitated complexes were undiluted or diluted 1:3 and 1:9 before the kinase assay as indicated. Quantification of the relative kinase activity of each complex is represented. (**b**) Protein extracts from *Cdk2*^−/−^ Lv141 and *Cdk2*^−/−^ Myc MEFs were separated by gel-filtration chromatography and distribution of cyclin A2, cyclin B1 and Cdk1 was analyzed by western blot (*indicates an unspecific band). (**c**) Cdk1 immunoprecipitation from fractions corresponding to free Cdk1 (18–21) and complexed cyclin A2/B1-Cdk1 (11–14) *in vitro* kinase assays on His-p27 as substrate. Relative levels of pT187-p27 vs p27 determined by signal densitometry are shown below each lane. (**d**) Proliferation rates of TKO and TKO-Myc MEFs measured by Crystal Violet staining after treatment with 10 µM of purvalanol A for 24, 48 and 72 hours. Error bars represent ± SD of three independent experiments. (**e**) Apoptotic rates of TKO and TKO Myc MEFs after treatment with 5 µM of Purvalanol A for 24 hours. Error bars represent ± SD of three independent experiments. Statistical analysis: T.test: *p < 0.1; **p < 0.05.

These results suggest that p27 proteasomal degradation mediated by the SCF^SKP2^ complex could be achieved even in the only presence of Cdk1. First, we verified that cells lacking Cdk2, Cdk4 and Cdk6 could efficiently phosphorylate p27 at the Thr-187 in TKO-Myc cells. TKO-Myc MEFs transfected with a p27-YFP construct showed phosphorylation at the Thr-187 of p27 (Fig. 8d). As control, TKO-Myc MEFs transfected with p27-YFP were treated with roscovitine, a Cdk2/Cdk1 inhibitor. After 6 hours of roscovitine treatment, pT187-p27 levels were highly reduced compared with that of untreated cells (Fig. 8d). Altogether, the results are consistent with the hypothesis that the phosphorylation is carried out by Cdk1. To corroborate this, Cdk1 immunocomplexes prepared from TKO and TKO-Myc MEFs were subjected to *in vitro* kinase assays using His-p27 as substrate. The results showed that overexpression of Myc (TKO-Myc cells) induced Cdk1-mediated p27 phosphorylation (Fig. 8e). To determine the cyclins involved in the regulation of Cdk1-mediated p27 phosphorylation in TKO-Myc cells we performed immunoprecipitations with antibodies for cyclin A2, cyclin B1and cyclin E as well as Cdk1 (Supplementary Fig. S6b) and determined their kinase activity on p27. Immunocomplexes of both cyclin A2 and cyclin B1 contained Cdk1 in TKO-Myc MEFs and were able to phosphorylate p27 *in vitro*. Immunoprecipitated cyclin E from TKO-Myc MEFs did not contain Cdk1 and consistently, showed no kinase activity (Fig. 9a). In order to determine which of the complexes (cyclin A2-Cdk1 or cyclinB1-Cdk1) was more active over p27 we carried out kinase assays with three different dilutions of the immunoprecipitated complexes (Fig. 9a). The kinase assays demonstrated that cyclin A2 and cyclin B1 immunocomplexes yielded similar p27 phosphorylation levels *in vitro*, although the amount of Cdk1 that co-immunoprecipitated with cyclin A2 was lower than did with cyclin B1. This result suggested that cyclin A2-Cdk1 complexes were more efficient in phosphorylating p27 at the Thr-187 than cyclin B1-Cdk1 complexes. However, complexes obtained by immunoprecipitating Cdk1 yielded p27 phosphorylation in a much lesser extent when compared to cyclin A2 and cyclin B1 immunocomplexes, being Cdk1 amounts much higher. To explain these results, we hypothesized that only a small fraction of the immunoprecipitated Cdk1 was complexed with cyclin A2 or cyclin B1, while the rest was unbound and thus, inactive Cdk1. To test this hypothesis, total protein lysates from C*dk2*^−/−^ Myc MEFs and their control *Cdk2*^−/−^ Lv141 MEFs were separated by gel filtration chromatography. The fractions containing Cdk1 were identified by western blot and the results showed that Cdk1 is distributed in high-molecular mass complexes corresponding to a molecular mass of about 80 kDa (Fig. 9b, fractions 11 to 14), and free form corresponding to a molecular mass of about 35 kDa, i.e., the molecular mass of Cdk1 (Fig. 9b, fractions 18 to 21). Cyclins B1 and A2 coeluted with Cdk1 in the high molecular mass fractions, which likely contain the cyclin-cdk complexes, but the amount of Cdk1 in the cyclin fraction is much lower than the free Cdk1 (Fig. 9b). We hypothesized that most of the Cdk1 immunoprecipitated was inactive because it was not bound to any cyclin. The fractions containing the free form of Cdk1 and the ones containing the high-molecular mass complexes of Cdk1, respectively, were pooled and Cdk1 was immunoprecipitated followed by *in vitro* kinase assay. These results showed that free-from Cdk1 was inactive while the Cdk1 from the high-molecular mass complexes showed kinase activity over p27 *in vitro* (Fig. 9c). These results revealed that most of the Cdk1 was unbound to cyclins, but as expected, only the cyclin-bound Cdk1 showed kinase activity on p27. Thus, we can attribute the low kinase activity of Cdk1 in Fig. 9 to a much higher ratio of inactive versus active Cdk1 present in the immunocomplexes obtained. Based on these data, we conclude that Myc can trigger p27 phosphorylation through the induction of Cdk1 activity independently of Cdk2 and Cdk4/6.

The synthetic lethality between Myc and Cdk1 has previously shown based on the selectivity of purvalanol against Cdk1^38,39^. We wanted to corroborate this in cells deficient in Cdk2, Cdk4 and Cdk6, as purvalanol A will target specifically Cdk1 in these cells. To do so, we compared the effect of purvalanol A on TKO and TKO-Myc cells. The results showed that purvalanol A inhibited more efficiently the growth of cells overexpressing Myc (Fig. 9d). TKO-Myc cells exposed to purvalanol A treatment showed a dramatic increase in annexin V binding compared with TKO control cells, indicating that Myc overexpression makes these cells prone to cell death in response to Cdk1 inhibition (Fig. 9e, Supplementary Fig. S7). The results corroborate the lethal synthesis between Cdk1 and Myc in this model.

### *Cdk1*-conditionally knock out cells verify that p27 phosphorylation can be mediated by cyclin B1-Cdk1 and induced by Myc

**Figure 10:**
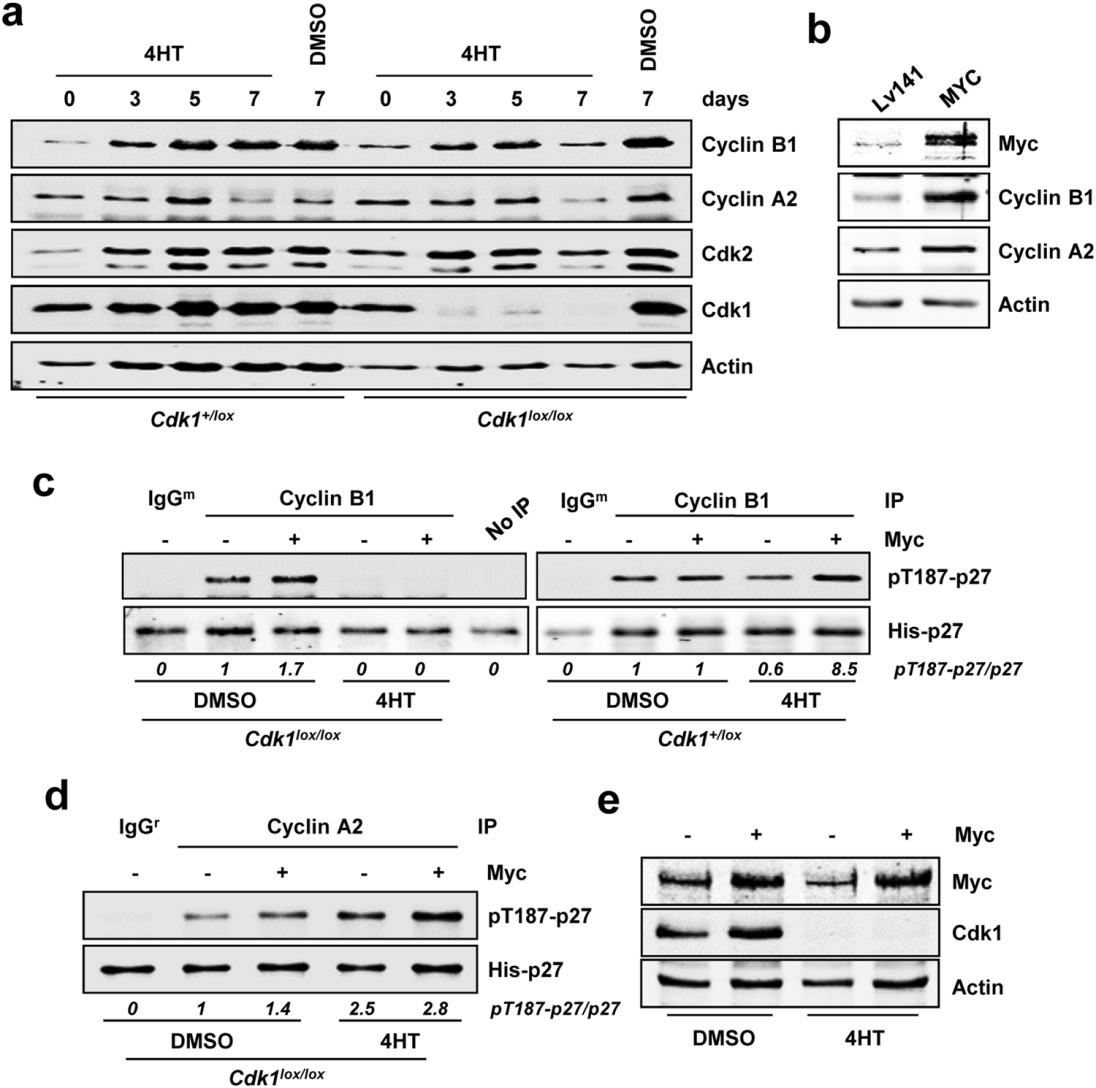
Conditional knock out of *Cdk1* totally abolishes cyclin B1-dependent phosphorylation of p27. (**a**) Protein extracts from *Cdk1* conditional knock out MEFs and controls treated with 0.6 μM 4HT or vehicle (DMSO) for different periods of time. Expression of cyclin A2, cyclin B1, Cdk1 and Cdk2 are shown. Actin levels were measured as loading control. (**b**) *Cdk1*^*lox/lox*^ MEFs transduced with a MYC-IRES-GFP construct or the corresponding empty vector. Myc overexpression assayed by western blot along with cyclin B1 and cyclin A2 levels. (**c**) *In vitro* kinase assay of cyclin B1 immunocomplexes on His-p27 in *Cdk1*^*lox/lox*^ and control *Cdk1*^+/*lox*^ treated with 4HT for 72 hours using p27 as substrate. (**d**) *In vitro* kinase assay of immunoprecipitated cyclin A2 from *Cdk1*^*lox/lox*^ MEFs transduced with a Myc-IRES-GFP construct or the corresponding empty vector and treated with 4HT for 72 hours. (**e**) Expression of Myc and Cdk1 in *Cdk1*^*lox/lox*^ MEFs transduced with a Myc-IRES-GFP construct and treated with 0.6 μM 4HT or DMSO for 72 hours. Actin levels were determined as loading control. Signal densitometry quantification of the kinase assays are shown below each lane. Kinase buffer with His-p27 was used as negative control (No IP) and unspecific IgG was used as negative control for the specificity of the antibody used for immunoprecipitation.

As described in the Introduction, Cdk1 has been reported as the only indispensable Cdk in animal cells, i.e., no other interphase Cdk is capable of supplying Cdk1 to accomplish an entire cell division^7^. To confirm the Cdk1-mediated phosphorylation of p27 at the Thr-187, we generated a Cdk1-conditional knock out MEF cell line (*Cdk1*^*lox/lox*^ MEFs) and its respective control (*Cdk1*^+/*lox*^ MEFs). Treatment of the *Cdk1*^*lox/lox*^ cell line with 4HT leads to *Cdk1* knock out, whereas the C*dk1*^+/*l*^*°*^*x*^ cell line shows normal Cdk1 expression (Fig. 10A). We first performed kinetics and found that after 72 hours of 4HT treatment, Cdk1 protein levels were almost undetectable, while control cells or cells treated with the vehicle (DMSO) did not show any change in Cdk1 levels (Fig. 10a). No major changes in Cdk2, cyclin A2 and B1 expression were found in Cdk1-depleted cells (Fig. 10a).

Next, the effect of Myc overexpression over Cdk1-mediated p27 phosphorylation was analyzed. For this purpose, cells were transiently transduced with a Myc-IRES-GFP construct or the corresponding empty vector (Lv141). Myc overexpression after 72 hours was confirmed by western blot and was accompanied with increased expression of cyclins A2 and B1 (Fig. 10b). We assayed the kinase activity on p27 of cyclin B1 immunoprecipitates from *Cdk1*^*lox/lox*^ MEFs and *Cdk1*^+/lox^ MEFs. In this immunoprecipitates, the kinase reaction should be carried out by cyclin B1-Cdk1 dimers, as cyclin B1-Cdk2 complexes have been reported but seem to be inactive or less efficient in the phosphorylation of RXL-containing substrates such as p27^40^. p27 phosphorylation was determined after 72 hours of 4HT or vehicle treatment and Myc-IRES-GFP transduction. Cyclin B1 complexes were able to phosphorylate p27 *in vitro*, in agreement with our previous results and those from others^29^ and its activity was increased upon Myc overexpression (Fig. 10c). Cdk1 depletion resulted in totally abolition of cyclin B1-associated kinase activity and Myc was not able to overcome it (Fig. 10c). This result demonstrates that cyclin B1-associated kinase activity over p27 is exclusively mediated through Cdk1, discarding the possibility that other Cdk could substitute for Cdk1 loss. Upon Myc overexpression, cyclin B1 complexes obtained from *Cdk1*^+/*lox*^ MEFs yielded higher pT187-p27 levels while Myc overexpression did not make any difference in 4HT-treated cells (Fig. 10c). Levels of immunoprecipitated cyclin B1 are shown in Supplementary Fig. S8. We also tested the effect of Myc overexpression on the kinase activity associated to cyclin A2 after Cdk1 ablation. The results showed that Myc overexpression led to higher cyclin A2-associated kinase activity on p27 in Cdk1-expressing cells (Fig. 10d). Moreover, depletion of Cdk1 expression resulted in enhanced cyclin A2-associated kinase activity, which was also increased upon Myc overexpression (Fig. 10d). This could be explained by a potencial increase in cyclin A2-Cdk2 complexes in the absence of Cdk1, suggesting that Cdk2 is more efficient in mediating p27 phosphorylation than Cdk1. The overexpression of Myc three days after lentiviral transduction and the ablation of Cdk1 in this experiment were assessed by western blot (Fig. 10e).

## Discussion

Our data unveils a novel mechanism by which Myc contributes to p27 degradation. It has been previously shown that *Skp2* is a Myc target gene and that, in order to be recognized by the ubiquitin ligase E3 complex SCF^SKP2^, p27 must be phosphorylated in Thr-187. This phosphorylation was previously reported to be carried out *in vitro* mainly by the heterocomplex cyclin E-Cdk2^30,33^. However, in the present work we have confirmed that it can also be mediated by Cdk1.

Which is the major cyclin that regulates Cdk1-mediated p27 phosphorylation in response to Myc overexpression? We show here that Myc can induce expression of cyclins A, B and E. Among them, only cyclin A2 and cyclin B1 would activate Cdk1 to phosphorylate p27 in the absence of Cdk2. Taken together, our results in MEFs suggest that both cyclin A2 and cyclin B1 are contributing to p27 phosphorylation through Cdk1. This is consistent with previous reports describing that cyclin B1‐Cdk1 can phosphorylate p27 *in vitro*^29,36^ while this is the first evidence of cyclin A2-Cdk1-mediated p27 phosphorylation. Moreover, this interaction would be more relevant in terms of p27 recognition by the ligase complex SCF^SKP2^, as cyclin A2 (but not cyclins E or B) has been described to be essential for this interaction^41^. Nevertheless, when we compared the activity of cyclin A2 or cyclin B1 immunocomplexes with that of Cdk1 immunocomplexes in TKO cells, we found that complexes obtained by pulling-down Cdk1 were much less efficient in phosphorylating p27 than the others. This can be explained if most of the Cdk1 is unbound to cyclins, and thus inactive, and this is indeed the case as demonstrated by gel filtration assays. Finally, the Myc-Cdk1-p27 axis is not only operative in mouse fibroblasts but also confirmed in human myeloid leukemia cells with conditional expression of Myc and p27.

Taken together, the results shown in this work indicate that Myc induces the phosphorylation of p27 at Thr-187 through the activation of not only Cdk2 (as previously described) but also through Cdk1. Cyclin B1 has Cdk1 as the unique partner capable of phosphorylating p27 in agreement with the fact that Cdk1 is being reported to show functions that cannot be compensated by any other^7^. Furthermore, the kinase activity on p27 associated to cyclin A2 increased after Myc overexpression. In this case, p27 phosphorylation would be carried out by the combination of Cdk1 and Cdk2 activities in control cells, as they both are cyclin A2 partners. However, the complete ablation of Cdk1 would increase the amount of Cdk2 bound to cyclin A2 and this would lead to higher p27 phosphorylation levels, suggesting that Cdk2 is more efficient in phosphorylating p27 at the Thr-187 than Cdk1. This result agrees with the ones obtained in Fig. 1, in which the efficiency of Cdk2-mediated p27 phosphorylation is much higher than that of Cdk1.

**Figure 11:**
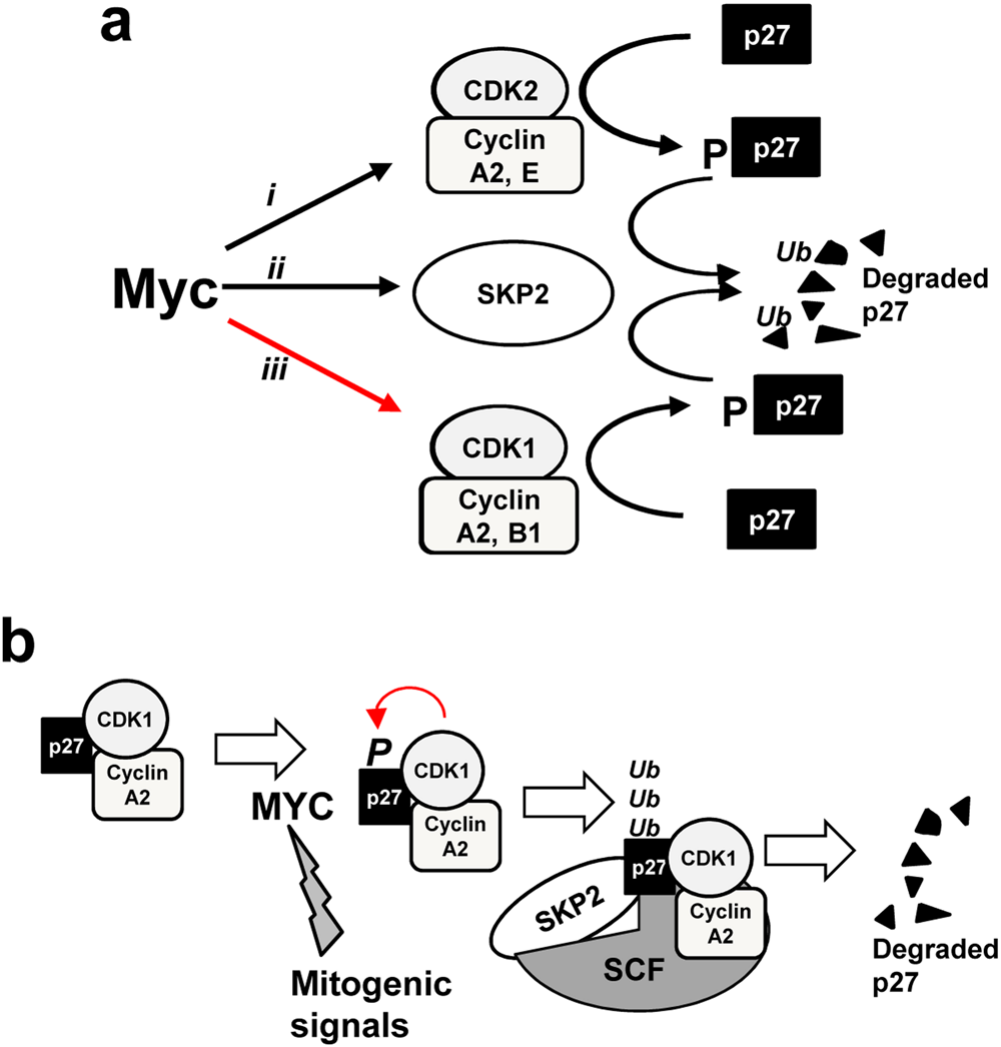
(**a**) Schematic model of the Myc pathways leading to p27 degradation. These are (i) the induction of Cdk2 activity; (ii) the induction of Skp2 and (iii) and the induction of Cdk1 activity, shown in this work. (**b**) Model of the effect of Myc on p27 degradation through p27 phosphorylation by Cdk1 and Skp2 induction. p27 can also be phosphorylated by Cdk2, not included in the figure.

In summary, Myc would promote p27 degradation through a triple effect: (i) the induction of Skp2 expression, which is the major pathway for p27 ubiquitination and degradation; (ii) the activation of Cdk2 via the upregulation of its cyclin partners and (iii) the activation of Cdk1, a mechanism previously unreported. These mechanisms are summarized in Fig. 11a. In tumor cells the high levels of Myc, or the induction of Myc levels by mitogenic stimuli in normal cells, would lead to p27 phosphorylation, ubiquitination and degradation. This pathway along the cell cycle is schematized in Fig. 11b. Synthetic lethality has been proposed as an alternative antitumoral therapy when the oncogene involved is not easily druggable. This would be the case of Myc^42^. The chemical inhibition of Cdk1 and Cdk2 has been previously identified to be synthetic-lethal with Myc or N-Myc overexpression^38,39,43^. As Myc’s pro-apoptotic effect under suboptimal conditions is well known^44^, it is conceivable that inhibition of Cdk1 would result in elevated p27 and thus an enhanced apoptotic effect of Myc. In conclusion, the results described here are relevant due to (i) the opposite correlation between Myc and p27 levels found in many human tumors and its correlation with poor disease outcome^45,46^ and (ii) the proposed new Myc-Cdk1 interaction involving p27 that could account for the still poorly understood mechanism that relies under the described synthetic lethality between Myc overexpression and Cdk1 pharmacological inhibition.

## Materials and Methods

### Cell culture

Mouse embryonic fibroblast (MEFs) cell lines were prepared from E12.5 embryos (from 129/svJXC57BL/6 J mice) following standard protocols^47^, in accordance with Spanish Animal Protection Law RD53/2013 and the European Directive ECC/566/2015, and with approval from the Institutional Animal Care and Use Committee (code 032-03) of the Centro Nacional de Investigaciones Oncológicas (CNIO). HeLa and HEK293T cell lines were from ATTC. MEFs, HeLa and HEK293T were grown in DMEM (Lonza). Kp27MER cell line derive from K562 (from ATCC) and was generated in the laboratory of the authors as described^19^ and was grown in RPMI (Lonza). All media were supplemented with 10% (v/v) fetal bovine serum (Lonza), 150 µg/ml gentamycin and 2 µg/ml ciprofloxacin, and additional antibiotics depending on the cell line, as described below. The following MEFs were transduced with the lentiviral vector Lv141-GFP-Myc or its respective Lv141-GFP empty vector to generate stably or transiently Myc-overexpressing cells: *Cdk2*^−/−^ MEFs (derived from *Cdk2*^−/−^ mice-G418 resistant); TKO MEFs (derived from *Ck2*^−/−^*;Cdk4*^−/−^*;Cdk6*^−/−^ mice-G418/puromycin/hygromycin resistant); *Ccne*^−/−^ MEFs (derived from *Ccne1/2*^−/−^ mice); *Cdk1*^*lox/lox*^ MEFs (derived from *Cdk1*^*lox/lox*^ mice) and *Cdk1*^+/*lox*^ MEFs (derived from *Cdk1*^+/*lox*^ mice). The following stably transfected or transduced cells were maintained in presence of the appropriate antibiotics: *Cdk2*^*−*/*−*^ MER MEFs were grown with 3 µg/ml of puromycin; pLKO-*Cdk2*^−/−^ MEFs and shCyclin A2-*Cdk2*^−/−^ MEFs were grown with puromycin 6 µg/ml; Kp27MER^19^ cells were grown with puromycin (0.5 µg/ml) and G418 (250 µg/ml). Cells were grown in normoxia in a humidified atmosphere at 37 °C and 5% CO_2_ except for TKO MEFs that were grown under hypoxia (3.5% O_2_). The plasmids used for transfection and lentiviral production are described in the Supplementary Table S1.

### Generation of Cdk1^lox^ mice

MEFs conditionally expressing Cdk1 were generated using the lox system. The targeting vector for generating the murine *Cdk1lox* allele was constructed by Gene Bridges (Heidelberg, Germany). In brief, the genomic region encoding *Cdk1* was cloned from the BAC RP23-178K6. The BAC-containing fragments of interest were then subcloned into a minimal pMV vector. The insertion of FRT-PGK-gb2-neo/km-FRT-loxP cassette as well as the distal loxP site into the *Cdk1* locus was performed by one Red/ET triple recombination step and verified by sequencing. The linearized targeting vector was electroporated into mouse embryonic stem cells (ES) by the Transgenic Unit of the CNIO (Madrid, Spain). ES cell clones having undergone proper homologous recombination were identified by southern blot. Single cell suspensions of two independent ES clones (ESSD12.188 and ESSD12.193) were microinjected into FVB donor blastocysts and implanted into hormonally induced pregnant C57BL/6 J females. Two male founder chimeras were utilized to generate germ-line transmission and crossed to a Flp-recombinase expressing strain to eliminate the PGK-gb2-neo selectable cassette. In the resulting conditional allele, the loxP sites flank exon 2 in the *Cdk1* recombinant locus. These mice were crossed with mice *Cre-ER*^+/+^ to generate *Cdk1*^*lox/lox*^; *CreER*^+/+^ mice. MEFs were prepared form E13.5 embryos. The CreER allele was generated as described^48^.

### Proliferation assays, treatments and transfections

For proliferation assays, 2.5 × 10^4^ cells/well were seeded in six-well plates, harvested when indicated and counted in a NucleoCounter apparatus (Chemometec). Alternatively, cell proliferation was measured by crystal violet staining. For quantification, crystal violet was solubilized with acetic acid measured at 595 nm absorbance as described^49^. Cell synchronization for cell cycle studies was carried out as follows: cells at a 60% confluence were washed with PBS and serum starved. After two days, media was removed, and fresh media containing 50 µM hydroxyurea was added and further incubated for 12 hours. Then, cells were washed with PBS and stimulated with DMEM supplemented with 10% FBS. Cells were harvested for RNA preparation and cell cycle assay when indicated. For cell cycle assay, one million of cells were resuspended with 3 mM EDTA-PBS and fixed with cold absolute ethanol overnight. Then, cells were incubated with Hoechst (5 µg/mL) for 30 min at 37 °C (protected from light) followed by cell cycle profile measurement in a MACSQuant VYB (Miltenyi Biotec). Results were analyzed with Flow Logic software (Miltenyi Biotec). Drugs were added when indicated at the following concentrations: 0.6 or 0.2 µM 4HT (4-hydroxi-tamoxifen) (Sigma-Aldrich) for MEFs or Kp27MER respectively, 30 µg/mL of cycloheximide (Sigma-Aldrich), 10 µM of roscovitine (Sigma-Aldrich), 50 µM of Zn_2_SO_4_ and 10 µM purvalanol A (Sigma-Aldrich). Transfection of MEFs was performed using PEI reagent (Polysciences, Inc). Briefly, PEI and DNA were mixed in free serum DMEM in a ratio 2.5:1 PEI:DNA (µg), vortexed and incubated 10–30 min RT. Culture media was replaced by half the amount of serum-free media and mixture of PEI + DNA was added to cells at 60–70% of confluence. After 12 hours of transfection mixture of PEI + DNA was removed, and cells supplemented with complete media. The list of constructs used in this work can be found in the online Supplementary Material (Supplementary Table S1).

### Lentivirus production and cell transduction

HEK293T cells were transfected with PEI as previously described to generate lentiviral particles. Mixture of packaging plasmids (pCMV-VSV-G and psPAX2 from Addgene) and construct of interest was performed in a ratio of 1:3:4 (VSV-G:psPAX2: lentiviral construct). Lentiviral particles-containing supernatants were collected and stored at 4 °C in two rounds (48 and 72 hours after transfection). Supernatants were clarified at 1500 rpm for 10 minutes and filtered. For lentivirus concentration, clarified supernatants were mixed with PEG8000 (final concentration of 15% PEG8000), homogenized by inversion and led at 4 °C for 24–72 h. The mixture was centrifuged at 1500*xg* for 30 minutes at 4 °C, and the pellet with lentivirus was dissolved in small volumes of serum-free media and stored at −80 °C. For lentivirus tittering, HeLa cells were infected with increasing amounts of concentrated lentiviral particles (usually from 0.1 to 10 µL) and selected with puromycin. A multiplicity of infection ≥5 was used to transduce MEFs. For infection, the cells were pelleted and resuspended in the corresponding volume of lentivirus with 8 µg/mL of polybrene. The mixture of cells and lentivirus was incubated at 37 °C for 1 hour, resuspended every 10 minutes and plated in half of the volume for the corresponding plate in serum-containing media. After 12–18 hours, the same volume of media was added to reach the final volume of the corresponding plate and 48 hours after transduction lentivirus-containing media was replaced by fresh media and puromycin selection when indicated.

### Annexin V binding assay

Cells were seeded in a 60% confluence and 12 h later treated with 5 µM purvalanol A for 24 h. Cells were harvested, washed twice with PBS-3 mM EDTA, resuspended in 100 µL of Binding Buffer (10 mM Hepes/NaOH, 140 mM NaCl and 2.5 mM CaCl_2_, pH 7.4) and 2 µL FITC Annexin V (BD Bioscience) and incubated for 30 min at 4 °C. Cells were washed twice with PBS and resuspended in 250 µL of PBS. Annexin V binding was assayed in MACSQuant VYB (Miltenyi Biotec) and the results were analyzed with Flow Logic software (Miltenyi Biotec).

### mRNA expression analysis by qPCR

RNA extraction was carried out using Tri Reagent (Sigma-Aldrich). Roughly, 5 × 10^5^ cells were lysed in 0.5 mL of Tri Reagent, 0.1 mL of chloroform was added and gently mixed. The mixture was incubated at RT for 2 minutes and centrifuged at 13000 rpm for 15 min. Nucleic acid containing aqueous phase was transferred to a new 1.5 mL tube. For RNA precipitation, 0.25 mL of isopropanol was added, mixed by inversion, incubated at RT for 10 minutes and centrifuged for 15 minutes at 13,000 rpm 4 °C. Supernatant was discarded and 0.5 mL of 70% ethanol added. The RNA containing pellet was mixed by vortexing and centrifuged for 5 minutes at 7500 rpm 4 °C. Supernatant was discarded, RNA air dried and resuspended in RNAse free water. RNA concentration was determined by using a NanoDrop and 200 ng were resolved in an agarose gel to check the integrity of the RNA. For cDNA conversion, the iScript cDNA Synthesis Kit from Bio-Rad was used following manufacturer’s protocol for 1 µg of RNA as template. SYBR Select Master Mix from Applied Biosystems was used to amplify cDNA in a CFX Connect Real-Time PCR Detection System from Bio-Rad. qPCRs were analyzed with the CFX Manager software. The mRNA expression of genes of interest was normalized to β-actin mRNA expression. Murine primer sequences used (forward and reverse primers in 5′→3′ direction) *Cdk1*: CGGCGAGTTCTTCACAGAG and AACCGGAGTGGAGTAACGAG; *Cdk4*:GGCCTGTGTCTATGGTCTGG and TTCAGCCACGGGTTCATATC; *Ccna2*:GCCAGCTGAGCTTAAAGAAAC and AACGTTCACTGGCTTGTCTTC; C*cnb1*: GACGTAGACGCAGATGATGG and GCCAGTCAATGAGGATAGCTC; *Ccne2*: GCATTCTGACCTGGAACCAC and GGAAGCAATGAACAATGAGG; *Skp2*: AATCTGCACCCAGACGTGAC and TGGAGCACTCGGACAGAATC; *bactin*: AGACTTCGAGCAGGAGATGG and AGTTTCATGGATGCCACAGG.

### Protein levels analysis by western blot

Roughly, 100 µl of 1% NP40 lysis buffer (50 mM Tris-HCl pH 8, 150 mM NaCl, 1 mM EDTA, 10 mM NaF, 1% NP40 (v/v), 0.1% SDS; protease (Calbiochem) and phosphatase (Sigma-Aldrich) inhibitors added immediately before use) were used to lyse 5 × 10^5^ cells. All the steps were performed at 4 °C. Adherent material was washed once with cold PBS and directly lysed in the plate using a scrapper and protein extracts were collected in a 1.5 mL tube. Suspension cells were pelleted, washed once with cold PBS and incubated in lysis buffer for 30 min on ice, mixing them every 10 min by pipetting. Protein samples were sonicated (10 cycles in a Bioruptor Plus Sonicator device) and finally clarify by centrifugation at 14,000 rpm for 20 min at 4 °C. The supernatant was transferred to a new tube and kept frozen until used. Protein quantification was carried out using the Qubit Protein Assay Kit in a Qubit 2.0 Fluorometer. Samples were resolved by SDS-PAGE and transferred to a nitrocellulose membrane. Blocking was carried out at room temperature using 4% BSA in TBS-T for 1 hour. Primary antibodies were used diluted in TBS-T 1%BSA at a final concentration of 1:1000 unless indicated. IRDye800 or lRDye680 (LiCor Biosciences) secondary antibodies or Trueblot anti-rabbit or anti-mouse IgG DyLight 800 secondary antibodies (Rockland) were used. Signals were recorded with and Odyssey Infrared Imaging Scanner (LiCor Biosciences). Quantification and densitometry analysis were carried out using the ImageJ software. Primary antibodies: cdc2 p34-(17) (Santa Cruz Biotechnology, sc-54, monoclonal antibody used for immunoprecipitations and western blots); cdc2 p34 C19 polyclonal (sc-954, used for western blots); cdk2 M2 (sc-163); cdk4 C22 (sc-260); cyclin A2 H432 (sc-239); cyclin B1 GNS1 (sc-245, used 1:500 in western blots); cyclin E H111 (sc-248, used 1:500); His-probe H15 (sc-813); myc N262 (sc-764, used 1:2000 5% BSA); p27 C19 (sc-528); p27 C19 (sc-528-G); skp2 p45 H435 (sc-7164); actin I19 (sc-1616); phospho-Cdk substrate motif (Cell Signaling, 9477), Phospho-Thr187 p27 (Invitrogen 71-7700, used 1:1000 in western blots); phospho-retinoblastoma (Ser780) Cell Signaling #9307, used 1:500 in western blots).

### Immunoprecipitation and *in vitro* kinase assays

Cell lysis was performed using of non-denaturing lysis buffer (50 mM Tri-HCl pH7.5, 150 mM NaCl, 1 mM EDTA, 0.5% NP40; protease (Calbiochem) and phosphatase (Sigma-Aldrich) inhibitors added immediately before use). After cell lysis and protein quantification as previously indicated, 50 µg of protein extracts were separated for whole cell extract analysis and 500 µg were used for each immunoprecipitation. Dynabeads-protein G (Invitrogen) were used to capture protein-antibody immunocomplexes. Briefly, Dynabeads (15 µL per IP) were washed twice with washing buffer (50 mM Tris-HCl pH 7.5, 150 mM NaCl, 1 mM EDTA, 0.5% NP40), added to the protein extract plus antibody mix and incubated overnight at 4 °C with rotation. Next, Dynabeads-protein G-immunocomplexes were collected using a magnet DynaMag (Invitrogen), washed four times with washing buffer and resuspended in 30 µL of 2x SDS-PAGE loading buffer. Protein-protein interactions were analyzed by western blot as previously described. Normal IgG was used as negative control for each immunoprecipitation reaction. Alternatively, 1/3 of Dynabeads-protein G-immunocomplexes were used for *in vitro* kinase assay. They were additionally washed twice with kinase buffer (50 mM Hepes-NaOH pH 7.2, 150 mM NaCl, 10 mM MgCl2, 2.5 mM EGTA, 1 mM EDTA, 1 mM DTT, 10% glycerol, 10 mM β-glycerophosphate and 10 mM NaF), resuspended in 30 µL kinase buffer supplemented with 50 µM ATP and 1 µg of recombinant His6-p27 (His-p27) protein and incubated for 30 min at 30 °C. The kinase reaction was stopped by adding 5xSDS-PAGE loading buffer to the reaction. Samples were boiled and phosphorylation of p27 at the Thr-187 was measured by western blot using a phospho-specific antibody anti phospho-Thr187 p27 (Invitrogen 71-7700). When indicated, the immunocomplexes were incubated with 10 µM purvalanol A (or DMSO as control) in the presence of ATP for 30 min at 30 °C. Afterwards, the His-p27 was added to each reaction, mixed, incubated for 30 min at 30 °C and analyzed as described. Normal IgG and kinase buffer supplemented with ATP and substrate were used as negative controls for the kinase reaction. The relative kinase activity was determined by densitometric quantification of the western blot using the ImageJ software and represented as the fraction of pT187-p27 signal relative to total p27.

### Gel-filtration chromatography of total protein extracts

10^7^ cells were lysed as described in 200–300 µL non-denaturing chromatography lysis buffer (50 mM Hepes-NaOH pH 7.2, 150 mM NaCl, 0.05% Triton X-100, 1 mM EDTA, 2.5 mM EGTA, 1 mM DTT, 10% glycerol and 1 mM PMSF (protease inhibitor, Calbiochem) and phosphatase (Sigma-Aldrich) inhibitors added immediately before use), incubated on ice for 30 min and protein extracts clarified at 14000 rpm for 20 min at 4 °C and transferred to a new tube. 200 µL of protein extracts were directly applied onto a Superdex 200 10/300 GLcolumn (GE Healthcare) pre-equilibrated with chromatography buffer (50 mM Hepes-NaOH pH 7.2, 150 mM NaCl, 0.05% Triton X-100, 1 mM EDTA, 2.5 mM EGTA, 1 mM DTT, 10% glycerol and 1 mM PMSF) and subjected to fast-performance liquid chromatography in an Äkta purifier apparatus (GE Healthcare) with a flow rate of 0.4 mL/min at 4 °C. The protein complexes eluting from the column with different volume retentions according to their molecular size were fractionated in 500 µL fractions. 40 µL of each collected fraction were mixed with 10 µL of 5X SDS-PAGE loading buffer and analyzed by western blot as indicated. Alternatively, the selected fractions containing the protein complexes were mixed and subjected to immunoprecipitation and *in vitro* kinase assay as previously described.

## Supplementary information

Supplementary information
