# Supplementary material for: Myc stimulates cell cycle progression through the activation of Cdk1 and phosphorylation of p27
Source: Sci Rep. 2019 Dec 10;9:18693. doi: 10.1038/s41598-019-54917-1 (PMC6904551; doi:10.1038/s41598-019-54917-1)
Supplement: Supplementary file 1 — Supplementary information [file 41598_2019_54917_MOESM1_ESM.pdf]

SUPPLEMENTARY MATERIAL

Myc stimulates cell cycle progression through the activation of Cdk1 and phosphorylation of p27

Lucía García-Gutiérrez[1,6], Gabriel Bretones[1,7], Ester Molina[1], Ignacio Arechaga[1], Catherine Symonds[3,8], Juan C. Acosta[2], Rosa Blanco[1], Adrián Fernandez[1], Leticia Alonso[1], Piotr Sicinski[4], Mariano Barbacid[3], David Santamaría[5], Javier León[1*]

[1]Instituto de Biomedicina y Biotecnología de Cantabria (IBBTEC), Universidad de Cantabria-CSIC, and Department of Molecular Biology, Universidad de Cantabria, Santander, Spain
[2]Edinburgh Cancer Research UK Centre, Institute of Genetics and Molecular Medicine, University of Edinburgh, Edinburgh, UK
[3]Experimental Oncology, Molecular Oncology Programme, Centro Nacional de Investigaciones Oncológicas (CNIO), Madrid, Spain
[4]Department of Cancer Biology, Dana-Farber Cancer Institute, Boston, USA
[5]University of Bordeaux, INSERM U1218, ACTION Laboratory, IECB, Pessac, France
[6]Present Address: Systems Biology Ireland, University College Dublin, Belfield, Dublin, Ireland.
[7]Present Address: Departamento de Bioquímica y Biología Molecular, Instituto Universitario de Oncología-IUOPA, Universidad de Oviedo, 33006 Oviedo, Spain
[8]Present Address: Global Oncology Franchise, EMD Serono, Rockland, Massachusetts, USA

*Corresponding author

Running Title: Myc activates Cdk1 to phosphorylate p27

Supplementary Table S1. List of plasmids used in this work.

Plasmid	Construct	Origin
**pEYFP**	Empty vector containing YFP protein	Clontech
**pEYFP-p27**	Human p27 fused with YFP	Our lab
**pCMV-Bam-HA-EV**	Empty vector	Addgene
**pCMV-cdc2-HA WT**	Cdk1 wild type gene tagged with HA	Addgene
**pCMV-cdc2-HA DN**	CDK1 D146N gene tagged with HA	Addgene
**EX-NEG-Lv141**	Lentiviral empty vector	Genecopoeia
**EX-Z2845-Lv141-MYC Flag**	Human Myc tagged with Flag; IRES GFP	Genecopoeia
**pLKO.1 control**	Lentiviral empty vector	SigmaMission
**pLKO.1 shMYC TRCN0000039640**	Lentiviral shRNA for human MYC gene	SigmaMission
**pLKO.1 shMYC TRCN0000039642**	Lentiviral shRNA for human MYC gene	SigmaMission
**pLKO.1 shMYC TRCN0000039655**	Lentiviral shRNA for human MYC gene	SigmaMission
**pLKO.1 shCyclin A2 TRCN00000**	Lentiviral shRNA for mousecyclin A2 gene	SigmaMission
**pCMV-VSV-G**	VSV-G gene encoding envelope lentiviral protein	Addgene
**psPAX2**	GAG and POL genes encoding packaging lentiviral proteins	Addgene

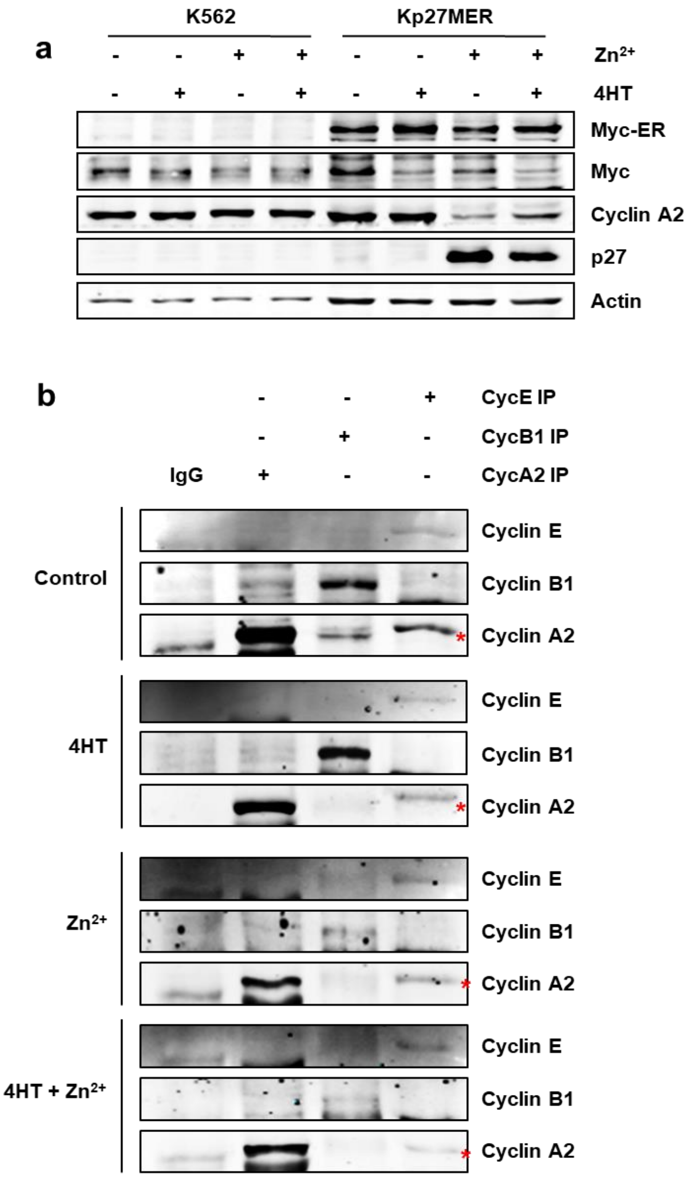

Supplementary Figure S1. Induction of p27 and activation of Myc-ER in Kp27MER cells. **(a)** Protein levels of Myc, Myc-ER, cyclin A2 and p27 of Kp27MER cells upon the indicated treatments for 24h hours compared with K562 parental cells. Actin levels were measured as loading control. **(b)** Levels of cyclins E, B1 and A2 after immunoprecipitation from Kp27MER treated as indicated for 24 hours. The asterisk marks a previous incubation with anti-cyclin E antibody.

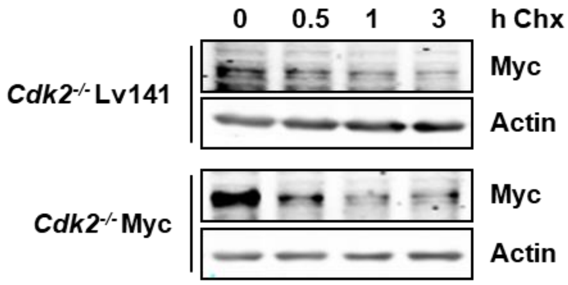

Supplementary Figure S2. Cycloheximide treatment of *Cdk2*$^{-/-}$ Lv141 or *Cdk2*$^{-/-}$ Myc MEFs leads to Myc rapid degradation. Protein stability of Myc as control of cycloheximide effectivity in *Cdk2*$^{-/-}$ Lv141 and *Cdk2*$^{-/-}$ Myc MEFs transfected with a p27-YFP construct measured by western blot. Levels of Myc were detected after 0, 0.5, 1 and 3 hours of cycloheximide treatment (30 µg/mL). Actin levels were used as loading control.

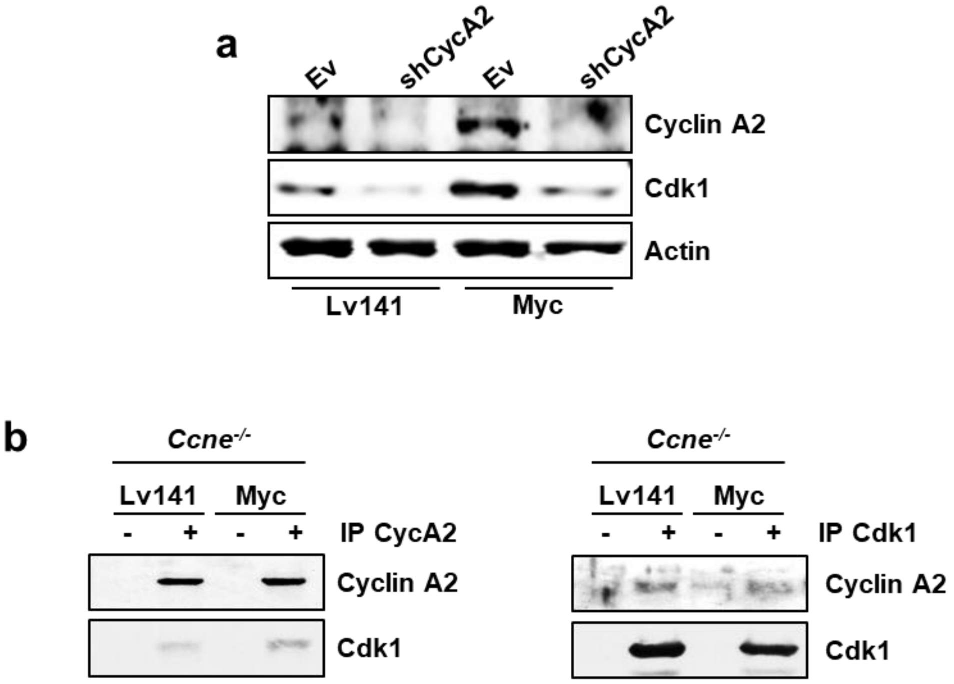

Supplementary Figure S3. Total lysates and immunoprecipitations from *Ccne*$^{-/-}$ Lv141 and *Ccne*$^{-/-}$ Myc MEFs. (a) Protein levels of cyclin A2 and Cdk1 from *Ccne*$^{-/-}$ Lv141 and *Ccne*$^{-/-}$ Myc MEFs transduced with shCyclin A2 lentiviral particles or the corresponding control (Ev). Actin levels were used as loading control. **(b)** Co-immunoprecipitation of cyclin A2 and Cdk1 in *Ccne*$^{-/-}$ Lv141 and *Ccne*$^{-/-}$ Myc MEFs. Unspecific IgG was used as negative control for the specificity of the antibody used for immunoprecipitation.

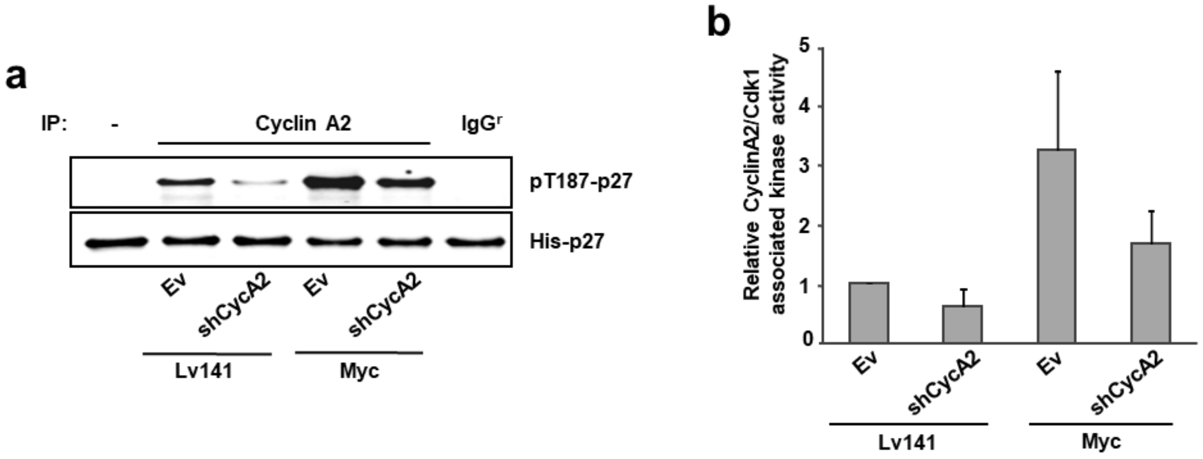

Supplementary Figure S4. Efficiency of cyclin A2 knock down in *Cdk2*$^{-/-}$ MEFs measured by its kinase activity over 27. (a) Kinase assay of cyclin A2 immunocomplexes obtained from *Cdk2*$^{-/-}$ Lv141 and *Cdk2*$^{-/-}$ Myc MEFs transduced with shCyclin A2 lentiviral particles or the corresponding control (Ev). **(b)** Densitometric quantification of the relative cyclin A2 kinase activity. Error bars represent ±SD of the quantification of three independent experiments. Kinase buffer with His-p27 was used as negative control (No IP) and unspecific IgG was used as negative control for the specificity of the antibody used for immunoprecipitation.

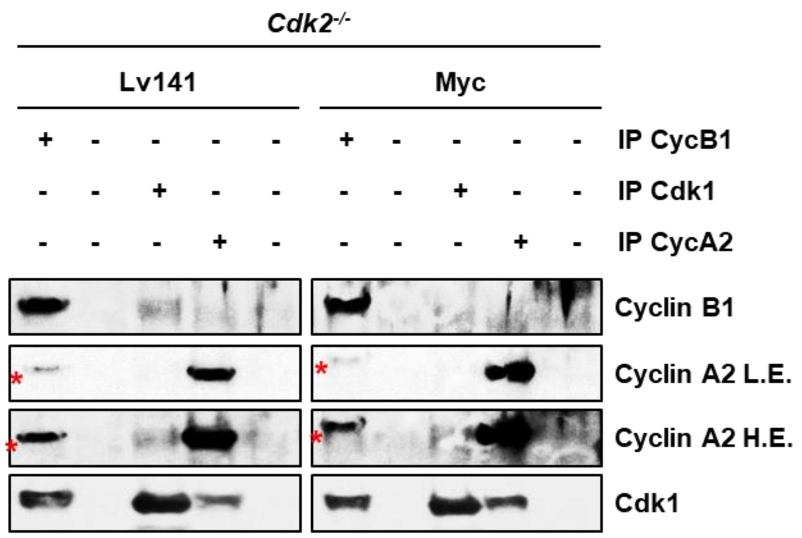

Supplementary Figure S5. Co-immunoprecipitation of cyclins A2 and B1 with Cdk1 in *Cdk2*$^{-/-}$ MEFs. Immunoprecipitated levels of cyclins A2 and B1 and Cdk1 from *Cdk2*$^{-/-}$ Lv141 and *Cdk2*$^{-/-}$ Myc MEFs. Unspecific IgG was used as negative control for the specificity of the antibody used for immunoprecipitation. The asterisk marks cyclin B1 from a previous incubation with anti-cyclin B1 antibody.

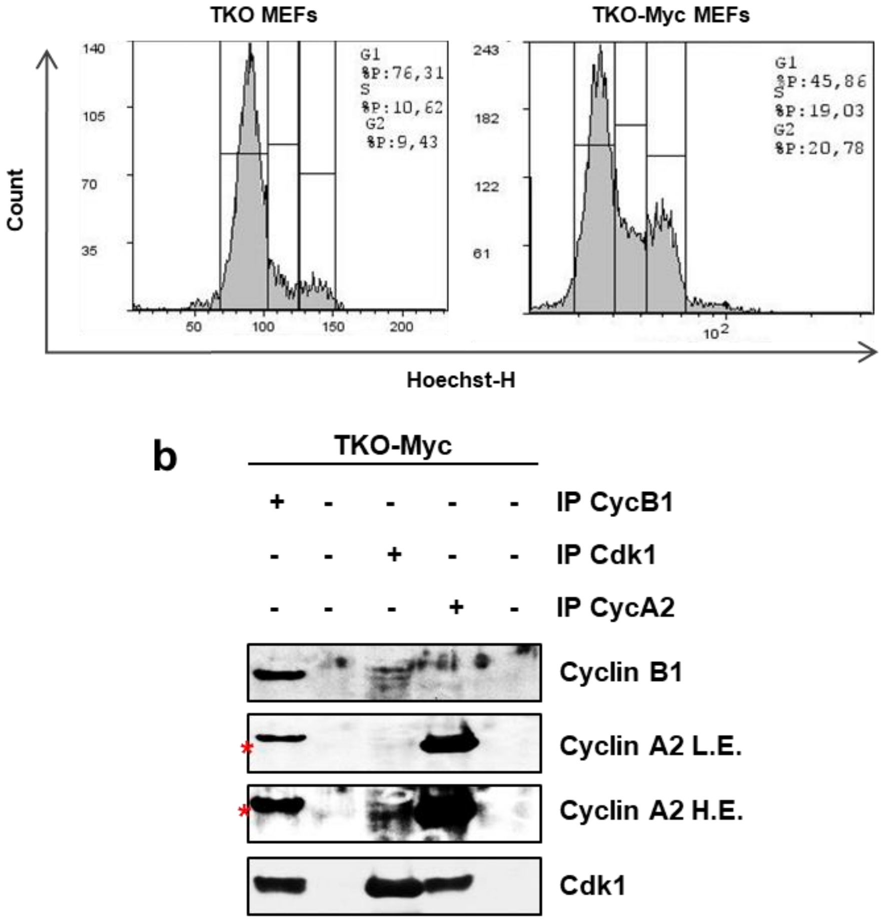

Supplementary Figure S6. Cell cycle profile of TKO and TKO-Myc MEFs and Cdk1 interaction with cyclins A2 and B1. (a) Distribution of cells among G_1, S and G_2 phases of the cycle of TKO and TKO-Myc MEFs. DNA content was measured using Hoechst staining by flow cytometry. **(b)** Immunoprecipitated levels of cyclins A2 and B1 and Cdk1 from TKO-Myc MEFs. Unspecific IgG was used as negative control for the specificity of the antibody used for immunoprecipitation. L.E, low exposure; H.L., high exposure. The asterisk marks cyclin B1 from a previous incubation with anti-cyclin B1 antibody.

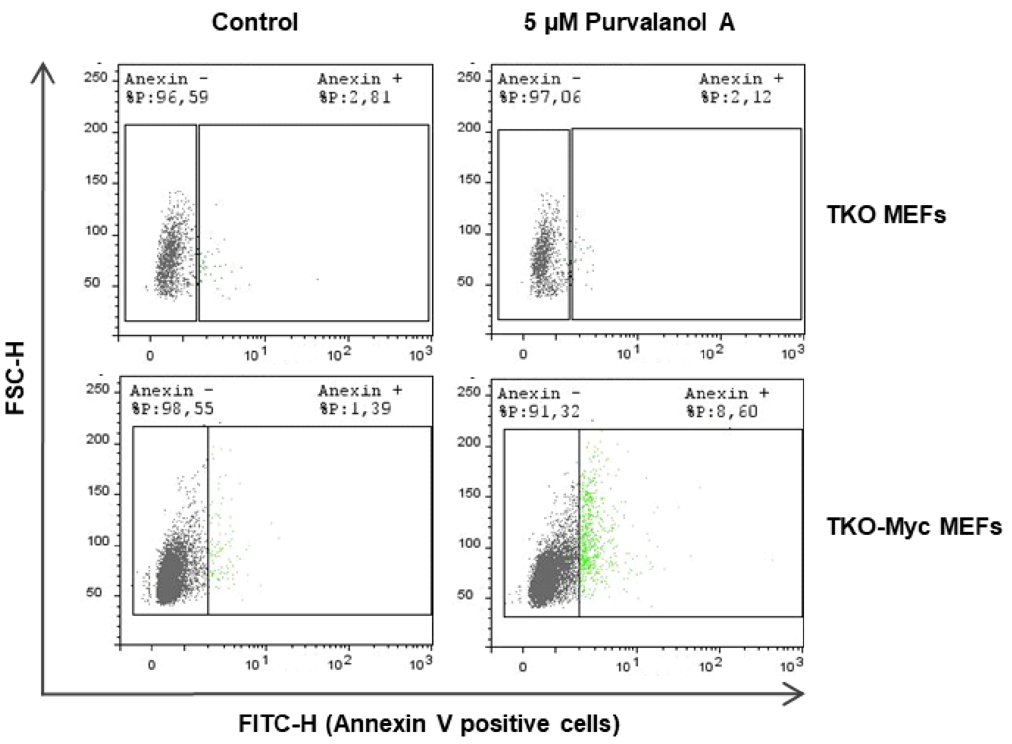

Supplementary Figure S7. Apoptotic assay measured by Annexin V positive cells. Original plots of Annexin V positive cells of TKO vs TKO-Myc cells treated with Purvalanol A for 24 h measured by flow cytometry.

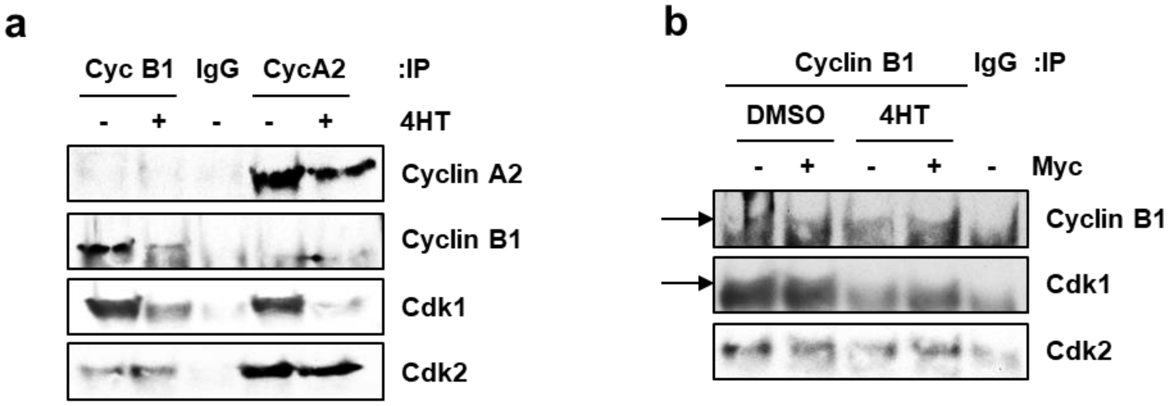

Supplementary Figure S8. Co-immunoprecipitation of cyclins A2 and B1 with Cdk1 and Cdk2 in *Cdk1*$^{lox/lox}$ MEFs. (a) Cyclin B1 and A2 immunoprecipitates from *Cdk1*$^{lox/lox}$ MEFs after treatment with 0.6 µM of 4HT or DMSO for 3 days. Levels of co-immunoprecipitated Cdk1 and Cdk2 are shown. **(b)** Myc overexpressing *Cdk1*$^{lox/lox}$ MEFs or their corresponding control cells treated for 3 days with 4HT 0.6 µM. Levels of cyclin B1, Cdk1 and Cdk2 after cyclin B1 immunoprecipitation are shown.
